# Supplementary material for: Topological network analysis of patient similarity for precision management of acute blood pressure in spinal cord injury
Source: eLife. 2021 Nov 16;10:e68015. doi: 10.7554/eLife.68015 (PMC8639149; doi:10.7554/eLife.68015)
Supplement: Supplementary file 1. [file elife-68015-supp1.zip › Fullcodepaper.html]

Supplementary code for: Topological network analysis of patient similarity for precision manAgement of acute blood pressure in spinal cord injury


# Supplementary code for: Topological network analysis of patient similarity for precision manAgement of acute blood pressure in spinal cord injury

#### A Torres-Espin, J Haefeli, R Ehsanian, D Torres, CA Almeida, JR Huie, A Chou, D Morozov, N Sanderson, B Dirlikov, CG Suen, JL Nielson, N Kyritsis, DD Hemmerle, JF Talbott, GT Manley, SS Dhall, WD Whetstone, JC Bresnahan, MS Beattie, SL McKenna, JZ Pan, AR Ferguson and the TRACK-SCI Investigators

#### 09/06/2021

# 1 Setting up

## 1.1 Libraries

Load the libreries used through the entire analysis

```
library(igraph) #to build and manAge graph data  
library(tidyverse) #data cleaning capabilities
library(reshape2) #data formatting
library(visNetwork) #for interactive netwrok visualization
library(vegan) #it implements a nice isomap interface
library(RColorBrewer) #for managing colors
library(TDAstats) #it implements persistent homology and other TDA methods
library(cccd) #to compute the k-NN graph
library(table1)#to create the demographic table
library(glmnet) #to fit the LASSO
library(glmnetUtils) #to fit the LASSO
library(caret) #for cross-validate the logistic regression
library(splines) #to fit the spline models
library(boot) ## for estimating LOOCV in glm models
library(viridis) ## color schema
library(ROCR) ## ROC processing
library(patchwork) ## for figures
library(png) ## for imAge processing for figures
library(grid) ## for figures
library(webshot) ## to take a shot at the graphs for figures
library(glmulti) ## for model selection
```

## 1.2 Custom helper functions

This script contains the custom functions used as helpers for the paper. Please note these functions are specific to the paper and have been coded to run with the main script of the publication. Running them in different settings might fail or produce unwanted results.

**plot\_legend:** Custom function for plotting the legends of the networks

```
plot_legend<-function(values, colors, legend_title, breaks, labels){
  #Custom function for plotting the legends of the networks
  #
  #@param values: values to be plotted
  #@param colors: the vector of colors to plot
  #@param legend_title: the string value for the legend title
  #@param breaks: the numeric vector for the x axis breaks
  #@param labels: the string vector for the x axis breaks
  #
  #@returns ggplot object with network gradient legend
  
  if (!is.numeric(values)){
    value<-as.numeric(as.factor(values))
  }else{
    value<-values
  }
  # plot(value, y=rep(0.5, length(value)),yaxt="n", ylab = '',
  #      type='n',frame.plot = F, xlim=c(min(value),max(value)))
  # legend_imAge <- as.raster(matrix(colors, nrow=1))
  # rasterImAge(legend_imAge, min(value), 0.3, max(value),0.5)
  
  
  # values<-mapping$mean.map
  # color<-net.color.Pal(250)
  # legend_title<-"MAP (mmHg)"
  df<-data.frame(values=seq(min(values), max(values), length.out = length(colors)))
  legend_plot<-ggplot(df, aes(values, y=1, fill=values))+
    geom_tile()+
    scale_fill_gradientn(colours = colors)+
    scale_x_continuous(breaks = breaks, labels = labels)+
    ggtitle(legend_title)+
    theme_minimal()+
    theme(plot.title = element_text(hjust = 0.5), axis.text.y = element_blank(),
          axis.title = element_blank(),
          axis.ticks.x = element_line(color="black"),
          axis.ticks.length.x = unit(3,"mm"),
          legend.position = "none",
          panel.grid = element_blank())
  
  return(legend_plot)
}
```

Back to top

**extract\_cumfreq:** Function to extract the cummulative frequency distribution for each patient by binning. The bin width is specified by the per\_step argument determining the percentAge respect the range of the specific measure the bin will cover. 1% by default

```
extract_cumfreq<-function(df,per_step=1){
  
  # Function to extract the cummulative frequency distribution for each patient by binning. The bin width is specified by the per_step argument determining the percentAge respect the range of the specific measure the bin will cover. 1% by default
  
  #@param df: the dataframe to extract the cummulative frequency dist in percentAge step
  #@param per_step: the percentAge width of binning
  
  fmin<-floor(min(df, na.rm = T))
  fmax<-ceiling(max(df, na.rm = T))
  step<-per_step*(fmax-fmin)/100
  range.t<-seq(fmin,fmax,step)
  
  cumfreq_int<-function(x, range.int=range.t){
    temp<-table(cut(as.numeric(x), breaks = range.t))
    temp<-temp/sum(temp)
    return(cumsum(temp))
  }
  
  result<-as.matrix(t(apply(df, 1, cumfreq_int)))
  
  return(result)
}
```

Back to top

**phom.dist2:** Wrap function of the phom.dist function from the TDAstats packAge to perform the wasserstein distance with power of 2 (see TDAstats packAge for details)

```
phom.dist2<-function (phom1, phom2){
  
#   Wrap function of the phom.dist function from the TDAstats packAge
# to perform the wasserstein distance with power of 2 (see TDAstats packAge for details)
  
  max.dim1 <- max(phom1[, 1])
  max.dim2 <- max(phom2[, 1])
  TDAstats:::wass_mat_calc(phom1, phom2, pow.val = 2, dim = max(max.dim1, 
                                                                max.dim2))
}
```

Back to top

**Net.viz:** This function plots the network. VisNetwork is an interface to vis.js packAge in JavaScript. The randomSeed of the network is established for reproducibility proposes so that we always get the same network shape.

```
Net.viz<-function(colors=NULL, network_name="", shot=T){
  
  # This function plots the network. VisNetwork is an interface to vis.js packAge in JavaScript. The randomSeed of the network is established for reproducibility proposes so that we always get the same network shape.
  
  #@param colors: colors to use in the network
  #@param network_name: name of the network to save file snapshot
  
  g.data$nodes$color<-colors
  net_vis<-visNetwork(nodes = g.data$nodes, edges = g.data$edges, height = "800px", width = "800px")%>%
    visIgraphLayout(randomSeed = 78,smooth = F, physics = T,
                    layout='layout_with_fr')%>%
    visEdges(color = list('inherit'='both', 'opacity'=0.5), width = 10)%>%
    visNodes(size=25, mass=3)%>%
    visPhysics(stabilization = T)
  
  if (shot){
    ## Save a png version of the network
    html_name <- tempfile(fileext = ".html") #set temporary file
    visSave(net_vis, html_name) #save the html network to temporary file
    webshot(html_name,file = paste0(network_name,".png"), delay = 1,
          vwidth = 800, vheight = 800,cliprect = c(80,100,700,700)) #webshot the html network
    # dev.off()
  }
  
  return(net_vis)
}
```

Back to top

**cNet.viz:** Visualize the contracted network. The CNet.viz function will plot the network

```
cNet.viz<-function(colors=NULL, network_name=""){
  
  #Visualize the contracted network. The CNet.viz function will plot the network
  
  #@param colors: colors to use in the network
  #@param network_name: name of the network to save file snapshot
  
  gcon.data$nodes$color<-colors
  net_vis_con<-visNetwork(nodes = gcon.data$nodes, edges = gcon.data$edges, height = "800px", width = "800px")%>%
    visIgraphLayout(randomSeed = 74,smooth = F, physics = T,
                    layout='layout_with_fr')%>%
    visEdges(color = list('inherit'='both', 'opacity'=0.5),length = 50, width = 3)%>%
    visNodes(scaling = list('max'=35, 'min'=10), mass=1.5)%>%
    visPhysics(stabilization = T)
  
  ## Save a png version of the network
  html_name <- tempfile(fileext = ".html") #set temporary file
  visSave(net_vis_con, html_name) #save the html network to temporary file
  webshot(html_name,file = paste0(network_name,"_con.png"), delay = 1,
        vwidth = 800, vheight = 800,cliprect = c(260,240,410,360)) #webshot the html network
  # dev.off()
  return(net_vis_con)
}
```

Back to top

**plot\_net:** Following function is used to plot the network plots in the paper and cluster plots with the specified variable and aggregating function (mean, median or proportion)

```
plot_net<-function(var, aggre.fun, y_axe_text, value.proportion,
                   prop.colors, prop.labels=c("A","B","C","D","E","NA"),
                   ranges=c(1,100), network_name){
  
  #Following function is used to plot the network plots in the paper and cluster plots with the specified variable and aggregating function (mean, median or proportion)
  
  #@param var: the variable to map onto the network
  #@param aggre.fun: the aggregated function to use: "mean", "median", "proportion"
  #@param y_axe_text: the text label in y axis to plot
  #@param value.proportion: value to compute the proportion for.
  #@param prop.colors: sequence of colors for the proportions
  #@param prop.labels: labels for the ggplot legend when aggre.fun = "proportion"
  #@param ranges:
  #@param network_name: name of the network to save file snapshot

  proportions<-function(x, ...){sum(x==value.proportion, na.rm=T)/length(na.omit(x))}
  
  if (ranges[1]!=1|ranges[2]!=100){
    values<-as.numeric(mapping[,var])
    
    minimal<-min(values, na.rm = T)+min(values, na.rm = T)*(ranges[1]/100)
    maximal<-max(values,na.rm = T)*(ranges[2]/100)
    values2<-ifelse(values<minimal, minimal, values)
    values2<-ifelse(values2>maximal, maximal, values2)
    values<-values2
  }else{
    values<-mapping[,var]
  }
  
  agg_cluster<-lapply(split(values, clusters$membership), get(aggre.fun), na.rm=T)
  agg_cluster2<-lapply(split(values, mapping$cluster), get(aggre.fun),na.rm=T)
  
  agg_cluster<-unlist(agg_cluster)
  agg_cluster2<-unlist(agg_cluster2)
  node.col.agg <- net.color.Pal(250)[as.numeric(cut(agg_cluster,breaks = 250))]
  node.col2.agg <- net.color.Pal(250)[as.numeric(cut(agg_cluster2,breaks = 250))]
  
  
  if(aggre.fun=='proportions'){
    map_colors<-as.numeric(mapping[,var])
    map_colors[is.na(map_colors)]<-which(prop.labels=="NA")
    node.col<-prop.colors[map_colors]
  }else{
    node.col<-net.color.Pal(250)[as.numeric(cut(values,breaks = 250))] 
  }
  
  net<-Net.viz(node.col, network_name) #replot the similarity network with the mapping var
  cnet<-cNet.viz(node.col.agg, network_name) #replot the contracted network with the mapping var
  
  if (aggre.fun%in%c('mean','median')){
    plot_c<-ggplot(mapping, aes_string('cluster', var,fill='cluster'))+
      geom_boxplot(size=0.6, outlier.size = 1.5)+
      scale_fill_manual(values=node.col2.agg)+
      theme_minimal(base_size = 11)+
      xlab('Patient cluster')+ylab(y_axe_text)+
      theme(legend.position = 'none', axis.text.x = element_text(size = 8))+
      scale_x_discrete(labels=paste('C', 1:11, sep=''))
  }else if(aggre.fun=='proportions'){
    prop.df<-mapping
    prop.df[,var]<-as.factor(prop.df[,var])
    plot_c<-ggplot(prop.df,aes_string('cluster', fill=var))+
      geom_bar(position='fill')+
      scale_fill_manual(values=prop.colors, na.value='grey50',
                        labels=prop.labels)+
      theme_minimal(base_size = 11)+
      xlab('Patient cluster')+ylab(y_axe_text)+
      theme(legend.position = 'none', 
            panel.grid.minor = element_blank(), 
            panel.grid.major.x = element_blank())+
      theme(legend.position = 'bottom', 
            legend.title = element_blank(),
            legend.key.size = unit(4, "mm"),
            legend.box = "horizontal",
            axis.text.x = element_text(size = 8))+
      guides(fill=guide_legend(nrow=1,byrow=TRUE))+
      scale_x_discrete(labels=paste('C', 1:11, sep=''))
  }
  
  return(list(net=net,con.net=cnet, plot=plot_c, 
              agg_values=agg_cluster,network_name=network_name))
}
```

Back to top

**prediction\_fun**: function to extract prediction metrics and prediction threshold

```
prediction_fun<-function(prob_prediction, real_class, ref){
  ## Function to extract LOOCV prediction metrics and prediction threshold
  #
  #@param prob_prediction: probability of prediction
  #@param real_class: the class to predict
  
  predictions<-prediction(prob_prediction, real_class)
  
  #Extract sensitivity and specificity into a dataframe
  roc_df<- data.frame(x=unlist(performance(predictions, "sens")@x.values),
                      sens_y=unlist(performance(predictions, "sens")@y.values),
                      spec_y=unlist(performance(predictions, "spec")@y.values))
  
  #calculate threshold as the point that balance sensitivity and specificity
  roc_df<-roc_df%>%
    mutate(diff_y = abs(sens_y-spec_y))
  
  #best prediction threshold
  pred_threshold<-roc_df$x[which.min(roc_df$diff_y)]
  
  #Predicting with the best threshold
  prediction_best<-ifelse(prob_prediction>pred_threshold, 1,0)
  conf_mat<-caret::confusionMatrix(reference=factor(real_class),
                                   data=factor(prediction_best), positive=ref)
  
  #Sensitivity-Specificity plot
  sense_spec_plot<-ggplot(roc_df, aes(x, sens_y))+
    geom_line(color="orange")+
    geom_line(data=roc_df, aes(x, spec_y), color = "steelblue")+
    geom_vline(xintercept = pred_threshold, linetype="dashed", 
               color="red", alpha=0.5)+
    theme_minimal()+
    ylab("Value (spec or sense)")+
    xlab("Prediction threshold")+
    annotate("text", label=round(pred_threshold, 3), x=0.55, y=0.1)+
    annotate("text", label="Sensitivity", x=0.1, y=0.85, color="orange")+
    annotate("text", label="Specificity", x=0.8, y=0.85, color="steelblue")+
    xlim(c(0,1))
  
  #AUC
  auc<-unlist(performance(predictions, "auc")@y.values)
  
  return(list("conf_mat"=conf_mat, "sense_spec_plot"=sense_spec_plot, 
              "auc"=auc, "roc_df"=roc_df))
}
```

Back to top

## 1.3 global settings

```
set.seed(666) #set a random seed for reproducibility of the random iterations in the code

# set color palette color-blind 'proof' for some graphs #from http://www.cookbook-r.com/Graphs/Colors_(ggplot2)/
cbbPalette <- c("#000000", "#E69F00", "#56B4E9","#009E73", "#F0E442", 
                "#0072B2", "#D55E00", "#CC79A7")

# Colors and pallet for the network plots
# net.color <- c('blue2','blue', 'green2','yellow','#ffa500','red2')
net.color <- plasma(6)
net.color.Pal <- colorRampPalette(net.color)
```

Back to top

# 2 Reading data

Reading files for both centers data and joining them. See paper. To reproduce the entire analysis and figures, make sure you download the dataset for ODC-SCI and specify the path.

```
## path to downloaded datasets
data1<-read.csv('../data/odc_sci_245.csv')
data2<-read.csv('../data/odc_sci_246.csv')

raw.df<-rbind(data1, data2)
colnames(raw.df)[1] <- "Subject_ID"

# raw.df<-read.csv('data/OR data publication.csv')
mapping<-raw.df
```

Back to top

# 3 Demographic table

Construct demographics table 1 in the paper

```
demographics.df<-raw.df[,c('Age', 'AIS_ad','AIS_dis', 'length_surg',
                           'AIS_is_improved','days_post_surgery_dish',
                           'Injury_level')]
demographics.df$AIS_is_improved<-as.character(demographics.df$AIS_is_improved)
demographics.df$AIS_is_improved[is.na(demographics.df$AIS_is_improved)]<-'N/A'
demographics.df$days_post_surgery_dish<-as.numeric(as.character(demographics.df$days_post_surgery_dish))
label(demographics.df$Age)<-'Age'
label(demographics.df$AIS_ad)<-'AIS admission'
label(demographics.df$AIS_dis)<-'AIS discharge'
label(demographics.df$length_surg)<-'Surgery duration'
label(demographics.df$days_post_surgery_dish)<-'Surgery to discharge'
label(demographics.df$AIS_is_improved)<-'AIS improvement'
label(demographics.df$Injury_level)<-'NLI admission'

units(demographics.df$Age)<-'years'
units(demographics.df$length_surg)<-'minutes'
units(demographics.df$days_post_surgery_dish)<-'days'

#Plot table 1
table1(~Age+AIS_ad+AIS_dis+length_surg+
         Injury_level+
         days_post_surgery_dish|AIS_is_improved, data=demographics.df, overall = F)
```

|  | N/A (N=15) | NO (N=61) | YES (N=42) |
| --- | --- | --- | --- |
| Age (years) |  |  |  |
| Mean (SD) | 46.0 (17.6) | 45.3 (19.1) | 51.4 (19.7) |
| Median [Min, Max] | 45.5 [19.0, 87.0] | 47.0 [18.0, 82.0] | 55.0 [18.0, 86.0] |
| Missing | 1 (6.7%) | 2 (3.3%) | 1 (2.4%) |
| AIS admission |  |  |  |
| A | 1 (6.7%) | 33 (54.1%) | 18 (42.9%) |
| B | 0 (0%) | 5 (8.2%) | 8 (19.0%) |
| C | 0 (0%) | 5 (8.2%) | 11 (26.2%) |
| D | 0 (0%) | 14 (23.0%) | 5 (11.9%) |
| E | 0 (0%) | 4 (6.6%) | 0 (0%) |
| Missing | 14 (93.3%) | 0 (0%) | 0 (0%) |
| AIS discharge |  |  |  |
| C | 1 (6.7%) | 4 (6.6%) | 15 (35.7%) |
| E | 1 (6.7%) | 2 (3.3%) | 5 (11.9%) |
| A | 0 (0%) | 35 (57.4%) | 0 (0%) |
| B | 0 (0%) | 5 (8.2%) | 5 (11.9%) |
| D | 0 (0%) | 14 (23.0%) | 17 (40.5%) |
| Missing | 13 (86.7%) | 1 (1.6%) | 0 (0%) |
| Surgery duration (minutes) |  |  |  |
| Mean (SD) | 433 (167) | 392 (146) | 407 (181) |
| Median [Min, Max] | 432 [121, 725] | 389 [120, 728] | 343 [151, 950] |
| Missing | 1 (6.7%) | 2 (3.3%) | 1 (2.4%) |
| NLI admission |  |  |  |
| Cervical | 2 (13.3%) | 36 (59.0%) | 33 (78.6%) |
| Other | 13 (86.7%) | 25 (41.0%) | 9 (21.4%) |
| Surgery to discharge (days) |  |  |  |
| Mean (SD) | 9.50 (2.12) | 18.8 (20.6) | 23.4 (23.8) |
| Median [Min, Max] | 9.50 [8.00, 11.0] | 11.0 [1.00, 128] | 14.5 [4.00, 120] |
| Missing | 13 (86.7%) | 4 (6.6%) | 2 (4.8%) |

## 3.1 Univariate test

Univariate test for demographics between improvers and non-improvers. T-test for numerical variables, Chi square test for categorical variables.

```
p.value.df<-demographics.df[demographics.df$AIS_is_improved!='N/A',]
p.value.df<-droplevels(p.value.df)
x<-p.value.df$AIS_is_improved

p.value.results<-list()
for (var in colnames(demographics.df)){
  y<-p.value.df[,var]
  if (isTRUE(all.equal(x,y))){next()}
  
  if (is.numeric(y)){
    p.value<-t.test(y~x)$p.value
  }else{
    p.value<-fisher.test(x,y)$p.value
  }
  p.value.results[[var]]<-p.value
}

p.value.results<-as.data.frame(do.call(rbind, p.value.results))

p.value.results
```

```
##                                  V1
## Age                    1.218759e-01
## AIS_ad                 1.371957e-02
## AIS_dis                6.355284e-11
## length_surg            6.605313e-01
## days_post_surgery_dish 3.290754e-01
## Injury_level           5.449344e-02
```

Back to top

# 4 Data preprocessing

This section performs the data preprocessing steps necessary to conduct the rest of the analysis.

- Extraction of the OR q5 values
- Extract the cummulative frequency distribution for each patient and measure

```
#Extraction of the OR q5 values
map.columns<-colnames(raw.df)[str_detect(colnames(raw.df), 'OR_MAP_Q')]
map.wide<-raw.df[,c('Subject_ID',map.columns)]

#Format in long for some applications
map_df_long<-melt(map.wide)

HR.columns<-colnames(raw.df)[str_detect(colnames(raw.df), 'OR_HeartRate_Q')]
HR.wide<-raw.df[,c('Subject_ID',HR.columns)]

#Format in long for some applications
hr_df_long<-melt(HR.wide)

#Extract the cumulative frequency distribution for each patient and measure
MAP.freq<-extract_cumfreq(map.wide[,-1],1) #the bin width is 1% of the MAP range
HR.freq<-extract_cumfreq(HR.wide[,-1],1) #the bin width is 1% of the HR range
```

**Table Example**

```
knitr::kable(map.wide[1:10,1:8])
```

| Subject\_ID | OR\_MAP\_Q0 | OR\_MAP\_Q5 | OR\_MAP\_Q10 | OR\_MAP\_Q15 | OR\_MAP\_Q20 | OR\_MAP\_Q25 | OR\_MAP\_Q30 |
| --- | --- | --- | --- | --- | --- | --- | --- |
| TSretro001 | NA | NA | NA | NA | NA | NA | 82.25 |
| TSretro002 | 81.00000 | 80.28 | 70.82 | 60.54 | 77.80000 | 68.50 | 68.81 |
| TSretro004 | NA | NA | NA | 73.97 | 52.46000 | 48.71 | 47.56 |
| TSretro005 | 87.20000 | 97.51 | 80.31 | 78.24 | 78.54000 | 78.95 | 79.45 |
| TSretro006 | NA | NA | NA | NA | 53.10588 | 55.01 | 53.82 |
| TSretro007 | 78.00000 | 65.91 | 68.48 | 71.17 | 68.38000 | 68.09 | 72.01 |
| TSretro009 | NA | NA | NA | NA | 69.12000 | 73.40 | 68.93 |
| TSretro010 | 77.40816 | 77.87 | 84.99 | 77.93 | 72.82000 | 77.42 | 79.90 |
| TSretro011 | NA | NA | NA | NA | NA | NA | 74.55 |
| TSretro012 | NA | NA | NA | 55.08 | 68.35000 | 61.85 | 65.86 |

## 4.1 Figure 1

Code for the construction of figure 1

```
## Load consort diagram
consort<-readPNG("or consort.png") ## This is an external imAge included as supplementary file. Make sure to specify the path location

consort_grob<-rasterGrob(consort)

or_map_labels<-c("OR_MAP_Q120","OR_MAP_Q480","OR_MAP_Q955")
or_HR_labels<-c("OR_HeartRate_Q120","OR_HeartRate_Q480","OR_HeartRate_Q955")

#MAP time plot
map_patient_plot<-ggplot(map_df_long, aes(x=variable, y=Subject_ID, fill=value))+
  geom_tile()+
  xlab('Time (Q5 minutes)')+ylab('SCI patients')+
  scale_fill_gradientn(colours = c('blue3','steelblue','lightgreen','orange','firebrick3'),
                       limits=c(40,140), na.value = 'grey')+
  scale_x_discrete(breaks=or_map_labels, labels=c('2h', '8h','16h'))+
  geom_vline(xintercept = c(24,96, 191), linetype=2)+
  labs(fill = "mmHg")+
  theme(legend.position = c(0.89,0.2), axis.text.y = element_blank(),
        axis.ticks.y = element_blank(),
        legend.background = element_blank(),
        legend.box.background = element_blank())

#plot mean MAP
map_patient_mean_plot<-ggplot(map_df_long, aes(x=Subject_ID, y=value, group=Subject_ID))+
  stat_summary(fun.y = 'mean', geom='bar', fill='orange', width=0.5)+
  stat_summary(fun.y = 'mean', geom='point')+
  coord_flip(ylim = c(55, 110))+
  theme(axis.text.y = element_blank(), axis.ticks.y = element_blank(),
        panel.grid = element_blank(), panel.background = element_blank())+
  ylab('aMAP (mmHg)')+xlab(NULL)+
  scale_y_continuous(breaks = c(60,80,100))

#HR time plot
hr_patient_plot<-ggplot(hr_df_long, aes(x=variable, y=Subject_ID, fill=value))+
  geom_tile()+
  xlab('Time (Q5 minutes)')+ylab('SCI patients')+
  scale_fill_gradientn(colors=c('yellow4','yellow','purple','purple4'),
                       values=c(0,0.2,0.5,0.9,1),na.value = 'grey',
                       limits=c(0,160))+
  scale_x_discrete(breaks=or_HR_labels, labels=c('2h', '8h','16h'))+
  geom_vline(xintercept = c(24,96, 191), linetype=2, alpha=0.6)+
    labs(fill = "bpm")+
  theme(legend.position = c(0.89,0.2), axis.text.y = element_blank(),
        axis.ticks.y = element_blank(),
        legend.background = element_blank(),
        legend.box.background = element_blank())

#Plot mean HR
hr_patient_mean_plot<-ggplot(hr_df_long, aes(x=Subject_ID, y=value))+
  stat_summary(fun.y = 'mean', geom='bar', fill='orange', width=0.5)+
  stat_summary(fun.y = 'mean', geom='point', fill='red')+
  coord_flip(ylim = c(50, 110))+
  theme(axis.text.y = element_blank(), axis.ticks.y = element_blank(),
        panel.grid = element_blank(), panel.background = element_blank())+
  ylab('aHR (beats per min.)')+xlab(NULL)+
  scale_y_continuous(breaks = c(60,80,100))

#Layout of the figure
layout <- c(
  #Line 1
  area(t=1, l=1, r=10, b=4),
  # Line 2
  area(t=5, l=1, r=3, b=7),area(t=5, l=4, r=5, b=7),area(t=5, l=6, r=8, b=7),
  area(t=5, l=9, r=10, b=7)
)

#Building the figure
figure1<-wrap_elements(consort_grob)+
  map_patient_plot+ggtitle("Mean Arterial Pressure (MAP)")+
  map_patient_mean_plot+
  hr_patient_plot+ggtitle("Heart Rate (HR)")+
  hr_patient_mean_plot

#Specify figure options
figure1<-figure1+plot_layout(design = layout)+
  plot_annotation(tag_levels = "a")

#Save the figure
ggsave(figure1,file="figure 1.png", width = 10, height = 12,dpi = "retina")

figure1
```

Back to top

## 4.2 Example plots

Example plots for visualizing the data and the preprocessing.

Generate the dataframe for ploting 4 raw examples and 4 processed examples (see paper for details)

```
#Raw map data for 4 patient examples
df.plot.raw<-data.frame(patient=c(rep('Patient 100', length(map.wide[100,-1])),
                                  rep('Patient 1', length(map.wide[1,-1])),
                                  rep('Patient 10', length(map.wide[10,-1])),
                                  rep('Patient 86', length(map.wide[86,-1]))),
                        map=c(unlist(map.wide[100,-1]),unlist(map.wide[1,-1]),
                              unlist(map.wide[10,-1]),unlist(map.wide[86,-1])),
                        q5=c(seq(1,length(map.wide[100,-1])),
                             seq(1, length(map.wide[1,-1])),
                             seq(1, length(map.wide[10,-1])),
                             seq(1, length(map.wide[86,-1]))))

# cummulative frequency distribution of map data for 4 patient examples
df.plot.cumfreq<-data.frame(patient=c(rep('Patient 100', length(MAP.freq[100,])),
                                      rep('Patient 1',length(MAP.freq[1,])),
                                      rep('Patient 10',length(MAP.freq[10,])),
                                      rep('Patient 86', length(MAP.freq[86,]))),
                            freq=c(unlist(MAP.freq[100,]), unlist(MAP.freq[1,]),
                                   unlist(MAP.freq[10,]),unlist(MAP.freq[86,])),
                            bin=c(1:length(MAP.freq[100,]), 1:length(MAP.freq[1,]),
                                  1:length(MAP.freq[10,]), 1:length(MAP.freq[86,])))

#Panel A
fig2_sup1_a<-ggplot(df.plot.raw,aes(q5, map, color=patient))+
  geom_line()+
  theme_minimal(base_size = 10)+
  ylab('MAP(mmHg)')+xlab('Time (Q5)')+
  theme(legend.title = element_blank(), legend.position = c(0.7, 0.9))+
  guides(color=guide_legend(nrow=2,byrow=TRUE))

#Panel B
fig2_sup1_b<-ggplot(df.plot.cumfreq,aes(bin, freq, color=patient))+
  geom_line()+
  theme_minimal(base_size = 10)+
  ylab('MAP cummulative\ndist. function')+xlab('Bin of MAP (1% binwidth)')+
  theme(legend.title = element_blank(), legend.position = c(0.8, 0.3))

fig2_sup1_a+fig2_sup1_b
```

Back to top

## 4.3 Optimization of isomap solutions

The following section iterates through the procedure to optimize the parameter selection for isomap according to the residual variance and the persistence homology criteria (see methods in paper for details)

```
#Combine MAP and HR cumulative freq. distributions. Each row is a patient
processed_df<-dist(cbind(MAP.freq,HR.freq), method = 'euclidean')

#'Iterates through the calculations of RV and wasserstein distances for
#'k 3 to 7 and dimensions kept from 1 to 12. (see methods in the paper for details)
opt.df<-list()
counter<-1
for (k in 3:7){
  gdist.k<-isomapdist(processed_df, k=k)
  ph.gdist.k<-calculate_homology(as.matrix(gdist.k),format = 'distmat')
  
  for (i in 1:12){
    isomap.k<-isomap(processed_df, k=k,ndim = i)
    ph.emd.k<-calculate_homology(as.matrix(dist(isomap.k$points)), 
                                 format = 'distmat')
    RV<-1-(cor(c(gdist.k), c(dist(isomap.k$points)))^2)
    WD<-phom.dist2(ph.gdist.k,ph.emd.k)
    opt.df[[counter]]<-data.frame(k=k, d=i, RV=RV,WD0=WD[1],WD1=WD[2])
    counter<-counter+1
  }
}
isomap_solution<-do.call(rbind, opt.df)

knitr::kable(isomap_solution)
```

|  | k | d | RV | WD0 | WD1 |
| --- | --- | --- | --- | --- | --- |
| 0 | 3 | 1 | 0.1894675 | 47.467251 | 36.633978 |
| 01 | 3 | 2 | 0.0278481 | 11.880598 | 10.865011 |
| 02 | 3 | 3 | 0.0231934 | 6.886587 | 7.325851 |
| 03 | 3 | 4 | 0.0147152 | 7.811643 | 6.448768 |
| 04 | 3 | 5 | 0.0139968 | 11.534617 | 5.422959 |
| 05 | 3 | 6 | 0.0134239 | 15.387671 | 4.628609 |
| 06 | 3 | 7 | 0.0131569 | 21.954244 | 3.692791 |
| 07 | 3 | 8 | 0.0140635 | 27.761348 | 3.719897 |
| 08 | 3 | 9 | 0.0143412 | 33.269958 | 3.668462 |
| 09 | 3 | 10 | 0.0143254 | 38.768747 | 3.281643 |
| 010 | 3 | 11 | 0.0147778 | 46.059926 | 3.959602 |
| 011 | 3 | 12 | 0.0150901 | 52.399398 | 4.677945 |
| 012 | 4 | 1 | 0.2749494 | 52.884087 | 25.067810 |
| 013 | 4 | 2 | 0.0358432 | 11.232465 | 9.386259 |
| 014 | 4 | 3 | 0.0285909 | 7.436811 | 9.578202 |
| 015 | 4 | 4 | 0.0201079 | 5.366194 | 7.714341 |
| 016 | 4 | 5 | 0.0178227 | 5.762252 | 6.690780 |
| 017 | 4 | 6 | 0.0184144 | 8.175809 | 6.029386 |
| 018 | 4 | 7 | 0.0178744 | 13.199958 | 4.544130 |
| 019 | 4 | 8 | 0.0179397 | 17.685910 | 3.962815 |
| 020 | 4 | 9 | 0.0181017 | 21.163906 | 4.064738 |
| 021 | 4 | 10 | 0.0194431 | 26.893813 | 5.283780 |
| 022 | 4 | 11 | 0.0192953 | 30.441103 | 5.285440 |
| 023 | 4 | 12 | 0.0197995 | 34.855487 | 5.326451 |
| 024 | 5 | 1 | 0.4103192 | 59.441069 | 16.589083 |
| 025 | 5 | 2 | 0.0407712 | 12.724088 | 6.983612 |
| 026 | 5 | 3 | 0.0340103 | 5.420716 | 5.349164 |
| 027 | 5 | 4 | 0.0319740 | 3.383266 | 5.268358 |
| 028 | 5 | 5 | 0.0274524 | 3.345140 | 4.206174 |
| 029 | 5 | 6 | 0.0240990 | 5.039918 | 3.906879 |
| 030 | 5 | 7 | 0.0228793 | 7.695615 | 3.289445 |
| 031 | 5 | 8 | 0.0224248 | 10.778404 | 3.710342 |
| 032 | 5 | 9 | 0.0231385 | 15.455183 | 3.551545 |
| 033 | 5 | 10 | 0.0227669 | 20.044685 | 2.897803 |
| 034 | 5 | 11 | 0.0226264 | 24.201491 | 3.198374 |
| 035 | 5 | 12 | 0.0229498 | 28.869162 | 3.583890 |
| 036 | 6 | 1 | 0.3992331 | 59.672703 | 11.639582 |
| 037 | 6 | 2 | 0.0334670 | 11.972590 | 3.875574 |
| 038 | 6 | 3 | 0.0289589 | 4.667536 | 3.111895 |
| 039 | 6 | 4 | 0.0247266 | 1.926340 | 1.633609 |
| 040 | 6 | 5 | 0.0225702 | 1.811851 | 1.262937 |
| 041 | 6 | 6 | 0.0207032 | 3.732140 | 1.217684 |
| 042 | 6 | 7 | 0.0203974 | 5.485051 | 1.760439 |
| 043 | 6 | 8 | 0.0204592 | 7.912305 | 1.660364 |
| 044 | 6 | 9 | 0.0200376 | 12.293921 | 1.180546 |
| 045 | 6 | 10 | 0.0205053 | 16.418045 | 1.219690 |
| 046 | 6 | 11 | 0.0205188 | 20.636482 | 1.216411 |
| 047 | 6 | 12 | 0.0198235 | 26.100459 | 1.012359 |
| 048 | 7 | 1 | 0.3952222 | 60.578793 | 8.340471 |
| 049 | 7 | 2 | 0.0374263 | 13.115918 | 2.906356 |
| 050 | 7 | 3 | 0.0309531 | 7.123999 | 2.832224 |
| 051 | 7 | 4 | 0.0260622 | 2.781431 | 1.959965 |
| 052 | 7 | 5 | 0.0240047 | 1.688020 | 2.283982 |
| 053 | 7 | 6 | 0.0214968 | 2.543622 | 2.000292 |
| 054 | 7 | 7 | 0.0197218 | 3.753766 | 1.648760 |
| 055 | 7 | 8 | 0.0199223 | 5.731631 | 1.815139 |
| 056 | 7 | 9 | 0.0198754 | 8.768754 | 2.032313 |
| 057 | 7 | 10 | 0.0189681 | 11.866291 | 1.500630 |
| 058 | 7 | 11 | 0.0191310 | 14.793726 | 1.385107 |
| 059 | 7 | 12 | 0.0192849 | 18.312078 | 1.404317 |

### 4.3.1 Optimization plots

```
# Plot RV, panel C
fig2_sup1_c<-ggplot(isomap_solution, aes(d, RV, color=factor(k), shape=factor(k)))+
  geom_point()+
  geom_line()+
  scale_x_continuous(breaks=1:12)+
  theme_minimal(base_size = 10)+
  theme(panel.grid.minor.x = element_blank(),
        legend.key.size = unit(0.4,'cm'),
        legend.position = c(0.8,0.7))+
  scale_color_manual(values=cbbPalette)+
  xlab('# dimensions kept')+ylab('Residual variance')+
  labs(color='Isomap k', shape='Isomap k')

# Plot WD 0 dimension, panel D
fig2_sup1_d<-ggplot(isomap_solution, aes(d, WD0, color=factor(k), shape=factor(k)))+
  geom_line()+
  geom_point()+
  scale_x_continuous(breaks = 1:12)+
  xlab('# dimensions kept')+ylab('Wasserstein 0-d distance')+
  theme_minimal(base_size = 10)+
  scale_color_manual(values=cbbPalette)+
  theme(panel.grid.minor = element_blank(), legend.position = "none")+
  labs(color='Isomap k', shape='Isomap k')

# Plot WD 1 dimension, panel E
fig2_sup1_e<-ggplot(isomap_solution, aes(d, WD1, color=factor(k), shape=factor(k)))+
  geom_line()+
  geom_point()+
  scale_x_continuous(breaks = 1:12)+
  xlab('# dimensions kept')+ylab('Wasserstein 1-d distance')+
  theme_minimal(base_size = 10)+
  scale_color_manual(values=cbbPalette)+
  theme(panel.grid.minor = element_blank(),legend.position = "none")+
  labs(color='Isomap k', shape='Isomap k')

fig2_sup1_c+fig2_sup1_d+fig2_sup1_e
```

Back to top

# 5 Exploratory network analysis suggests an upper and lower limit of intraoperative MAP for recovery

The following section of the code extracts the similarity network as specified in the paper.

## 5.1 Extract Network

```
iso.all<-isomap(processed_df,k=6, ndim = 4)#Solution from the isomap optimization
D<-iso.all$points

#' Iteration until to find the minimal k for K-nn mutual graph with 
#' one single component (each patient connected to at least one other)
k<-1
g<-nng(x=D, k = k, mutual = T)
g<-igraph::simplify(g)
g<-as.undirected(g)

while (max(components(g)$membership)>1) {
  k<-k+1
  g<-nng(x=D, k = k, mutual = T)
  g<-igraph::simplify(g)
  g<-as.undirected(g)
}

g<-igraph::simplify(g)#simplify the graph to eliminate self referencing edges
g<-add_layout_(g, nicely())#add layout to the graph (only for visualization using plot())
# plot(g)# raw plot of the graph (see igraph packAge for more details)
```

Back to top

## 5.2 Plot the ‘naked network’

```
g.data<-toVisNetworkData(g) #transform g into a VisNetwork object for visualization
Net.viz(shot=T)
```

```
if (!is.null(dev.list())) {
  dev.off()
}
```

```
## null device 
##           1
```

Back to top

## 5.3 Network clustering

The following code finds the community structure (clusters) using the walktrap algorithm.

```
clusters<-c()
member.temp<-list()
modularity.df<-c()

#iterates through an arbitrary range of randoms steps (from 1 to 100)
for (i in 1:100){
  g.clusters<-cluster_walktrap(g, steps = i)
  clusters[i]<-max(g.clusters$membership)
  member.temp[[i]]<-g.clusters$membership
  modularity.df[i]<-modularity(g.clusters,g.clusters$membership)
}

#Choose the solution that maximise modularity
steps.t<-which.max(modularity.df) 

#Construct data frame from the results for visualization
cluster.df<-data.frame(steps=c(1:length(clusters)), 
                       n_clusters=clusters, modu=modularity.df)
```

Back to top

### 5.3.1 Random walk plots

```
#Panel F
fig2_sup1_f<-ggplot(cluster.df, aes(x=steps, y=modu))+
  geom_line(color='blue')+
  xlab('Number of random steps')+
  ylab('Modularity (Q)')+
  theme_minimal(base_size = 10)+
  geom_vline(xintercept = steps.t, linetype='dashed')

# ggsave('modu-steps.png', dpi='print', height = 2, width = 2.4)

#Panel G
fig2_sup1_g<-ggplot(cluster.df, aes(x=steps, y=n_clusters))+
  geom_line(color='blue')+
  xlab('Number of random steps')+
  ylab('Number of clusters')+
  theme_minimal(base_size = 10)+
  geom_vline(xintercept = steps.t, linetype='dashed')

fig2_sup1_f+fig2_sup1_g
```

```
# ggsave('clustes-steps.png', dpi='print', height = 2, width = 2.4)
```

Back to top

## 5.4 Collapsed network

The following code extract the collapsed network based on the clustering

```
clusters<-cluster_walktrap(g, steps =steps.t)
gcon<-contract.vertices(g, clusters$membership,vertex.attr.comb=toString) #contract the network
gcon<-igraph::simplify(gcon)#Eliminate the self-referencing edges

gcon.data <- toVisNetworkData(gcon)
n_patients_cluster<-as.vector(table(clusters$membership)) #calculate number of patients per cluster

#Extraction of colors for the dendogram
cluster_colors<-brewer.pal(11, 'Paired')
```

### 5.4.1 Plot the dendogram

```
dendPlot(clusters, mode = 'p',direction='downwards',show.tip.label=F,
         palette = cluster_colors)
```

```
# save the plot
png("dendogram.png")
  dendPlot(clusters, mode = 'p',direction='downwards',show.tip.label=F,
         palette = cluster_colors)
dev.off()
```

```
## png 
##   2
```

Back to top

## 5.5 Network plots

The following section include the network plots.

```
#Here the labels for the clusters are override to those provided in the paper
mapping$cluster<-factor(clusters$membership)
levels(mapping$cluster)<-c(9,5,7,2,3,4,6,11,10,8,1)
mapping$cluster<-factor(mapping$cluster, levels=1:11)

gcon.data$nodes$label<-paste('C',c(9,5,7,2,3,4,6,11,10,8,1), sep='')
gcon.data$nodes$value<-n_patients_cluster*2 #Set the size of the node proportional to the number of patients on it
```

### 5.5.1 Extract mapped networks

This section creats the networks mapped to specified variables using the custom helper functions at the begining of this document.

```
#MAP net
MAP.gcon<-plot_net('mean.map','mean','AverAge MAP (mmHg)', network_name = "map_net")

#HR net
HR.gcon<-plot_net('mean.HR','mean','AverAge HR (bpm)', network_name = "HR_net")

# AIS improved
mapping$AIS_is_improved<-as.factor(mapping$AIS_is_improved)
mapping$AIS_dis<-as.factor(mapping$AIS_dis)
mapping$AIS_is_improved_num<-as.numeric(mapping$AIS_is_improved)
mapping$AIS_is_improved_num[mapping$AIS_is_improved_num==3]<-NA
mapping$AIS_dis_num<-as.numeric(mapping$AIS_dis)
#2 is the code for improvers, this tells the function to call proportion of improvers

AIS_is_improved.gcon<-plot_net(var='AIS_is_improved_num','proportions','# of patients',2,prop.colors=c('#0000EE', '#FF3030', '#7F7F7F'),network_name = "AIS_is_improved_net", prop.labels = c("No","Yes", "NA"))

#AIS at discharge
proportion_colors<-c('blue','#329ce5', 'green','#ffa500','red','grey50')

# Extract proportions of AIS discharge by cluster (A=1, E=5)
AIS_dis1<-plot_net(var='AIS_dis_num', 'proportions','# of patients',1,
                             prop.colors=proportion_colors,
                   prop.labels = c("A","B","C","D","E", "NA"),
                   network_name = "AIS_disA")
AIS_dis2<-plot_net('AIS_dis_num', 'proportions','# of patients',2,
                              prop.colors=proportion_colors,
                   prop.labels = c("A","B","C","D","E", "NA"),
                   network_name = "AIS_disB")
AIS_dis3<-plot_net('AIS_dis_num', 'proportions','# of patients',3,
                              prop.colors=proportion_colors,
                   prop.labels = c("A","B","C","D","E", "NA"),
                   network_name = "AIS_disC")
AIS_dis4<-plot_net('AIS_dis_num', 'proportions','# of patients',4,
                              prop.colors=proportion_colors,
                   prop.labels = c("A","B","C","D","E", "NA"),
                   network_name = "AIS_disD")
AIS_dis5<-plot_net('AIS_dis_num', 'proportions','# of patients',5,
                              prop.colors=proportion_colors,
                   prop.labels = c("A","B","C","D","E", "NA"),
                   network_name = "AIS_disE")
```

Back to top

**Data frame for aggregated cluster scatter plots**

```
# data frame for Scatter plots of the aggregated values for each cluster
agg_plot_df<-data.frame(map=MAP.gcon$agg_values, 
                        AIS=AIS_is_improved.gcon$agg_values,
                        HR=HR.gcon$agg_values,
                        AIS_dis_A=AIS_dis1$agg_values,
                        AIS_dis_B=AIS_dis2$agg_values,
                        AIS_dis_C=AIS_dis3$agg_values,
                        AIS_dis_D=AIS_dis4$agg_values,
                        AIS_dis_E=AIS_dis5$agg_values)

agg_MAP_net_plot<-ggplot(agg_plot_df, aes(map, AIS, fill=AIS))+
  geom_point(shape=21, size=3)+
  scale_fill_gradientn(colors=net.color)+
  xlab('Mean cluster AverAge MAP (mmHg)')+
  ylab('Proportion of\n AIS improve.')+labs(fill='Proportion\nAIS improve.')+
  theme_minimal(base_size = 11)+
  geom_smooth(span=1, se=F)

agg_HR_net_plot<-ggplot(agg_plot_df, aes(HR, AIS, fill=AIS))+
  geom_point(shape=21, size=3)+
  scale_fill_gradientn(colors=net.color)+
  xlab('Mean cluster AverAge HR (bpm)')+
  ylab('Proportion of\n AIS improve.')+labs(fill='Proportion\nAIS improve.')+
  theme_minimal(base_size = 11)+
  geom_smooth(span=1, se=F)

AIS_disA_plot<-ggplot(agg_plot_df, aes(map, AIS_dis_A, fill=AIS_dis_A))+
  geom_point(shape=21, size=2)+
  scale_fill_gradientn(colors=net.color)+
  xlab('Mean cluster AverAge MAP (mmHg)')+
  ylab('Dis. proportion of\n AIS A')+labs(fill='% AIS A')+
  theme_minimal(base_size = 11)+
  geom_smooth(span=1, se=F)

AIS_disB_plot<-ggplot(agg_plot_df, aes(map, AIS_dis_B, fill=AIS_dis_B))+
  geom_point(shape=21, size=2)+
  scale_fill_gradientn(colors=net.color)+
  xlab('Mean cluster AverAge MAP (mmHg)')+
  ylab('Dis. proportion of\n AIS B')+labs(fill='% AIS B')+
  theme_minimal(base_size = 11)+
  geom_smooth(span=1, se=F)

AIS_disC_plot<-ggplot(agg_plot_df, aes(map, AIS_dis_C, fill=AIS_dis_C))+
  geom_point(shape=21, size=2)+
  scale_fill_gradientn(colors=net.color)+
  xlab('Mean cluster AverAge MAP (mmHg)')+
  ylab('Dis. proportion of\n AIS C')+labs(fill='% AIS C')+
  theme_minimal(base_size = 11)+
  geom_smooth(span=1, se=F)

AIS_disD_plot<-ggplot(agg_plot_df, aes(map, AIS_dis_D, fill=AIS_dis_D))+
  geom_point(shape=21, size=2)+
  scale_fill_gradientn(colors=net.color)+
  xlab('Mean cluster AverAge MAP (mmHg)')+
  ylab('Dis. proportion of\n AIS D')+labs(fill='% AIS D')+
  theme_minimal(base_size = 11)+
  geom_smooth(span=1, se=F)

AIS_disE_plot<-ggplot(agg_plot_df, aes(map, AIS_dis_E, fill=AIS_dis_E))+
  geom_point(shape=21, size=2)+
  scale_fill_gradientn(colors=net.color)+
  xlab('Mean cluster AverAge MAP (mmHg)')+
  ylab('Dis. proportion of\n AIS E')+labs(fill='% AIS E')+
  theme_minimal(base_size = 11)+
  geom_smooth(span=1, se=F)
```

Back to top

### 5.5.2 Figure 2

```
## Flow

flow<-readPNG("Figure 2 flow.png") #external file added as supplement in the paper
flow<-wrap_elements(rasterGrob(flow, interpolate = T,
                                       width = unit(1, "native"), 
                       height = unit(1, "native")))

#Map net
map_net <- readPNG('map_net.png')
map_net_grob<-wrap_elements(rasterGrob(map_net, interpolate = T,
                                       width = unit(1, "native"), 
                       height = unit(1.2, "native")),clip = F)
#Map contracted net
map_net_con <- readPNG("map_net_con.png")
map_net_con_grob<-wrap_elements(rasterGrob(map_net_con, interpolate = T,
                                           width = unit(1, "native"), 
                       height = unit(1.2, "native")),clip = F)
#HR net
HR_net <- readPNG('HR_net.png')
HR_net_grob<-wrap_elements(rasterGrob(HR_net, interpolate = T,
                                      width = unit(1, "native"), 
                       height = unit(1.2, "native")), clip = F)
#HR contracted net
HR_net_con <- readPNG("HR_net_con.png")
HR_net_con_grob<-wrap_elements(rasterGrob(HR_net_con, interpolate = T,
                                          width = unit(1, "native"), 
                       height = unit(1.2, "native")),clip = F)

#AIS_is_improved net
AIS_is_improved_net <- readPNG('AIS_is_improved_net.png')
AIS_is_improved_net_grob<-wrap_elements(rasterGrob(AIS_is_improved_net, interpolate = T,
                                      width = unit(1, "native"), 
                       height = unit(1.2, "native")), clip = F)
#AIS_is_improved contracted net
AIS_is_improved_net_con <- readPNG("AIS_is_improved_net_con.png")
AIS_is_improved_net_con_grob<-wrap_elements(rasterGrob(AIS_is_improved_net_con, interpolate = T,width = unit(1, "native"), 
                       height = unit(1.2, "native")),clip = F)

#Legends
legend.map<-plot_legend(mapping$mean.map, net.color.Pal(250), "AverAge MAP\n(mmHg)", c(70, 85, 100), c("70", "85","100"))

legend.HR<-plot_legend(mapping$mean.HR, net.color.Pal(250), "AverAge HR\n(bpm)", c(60, 80, 100), c("60", "80","100"))

legend.AIS<-plot_legend(25:75, net.color.Pal(250), "Proportion\n AIS improve. (%)", c(25, 50, 75), c("<25", "50",">75"))

#temporal AIS ggplot to extract the legend
temp_AIS<-AIS_is_improved.gcon$plot+
  labs(fill="AIS improve.")+
  theme(legend.position = "right", 
        legend.title = element_text(), 
        legend.direction = "vertical")+
  guides(fill=guide_legend(nrow=3,byrow=TRUE))

legend_AIS_cat<-wrap_elements(ggplotGrob(temp_AIS)$grobs[[15]])

#Build figure
figure<-flow+
  map_net_grob+
  map_net_con_grob+inset_element(legend.map, left=-0.2, bottom = -0.1, 
                                   right = 0.3, top=0.4, ignore_tag = T)+
  MAP.gcon$plot+
  HR_net_grob+
  HR_net_con_grob+inset_element(legend.HR, left=-0.2, bottom = -0.1, 
                                   right = 0.3, top=0.4, ignore_tag = T)+
  HR.gcon$plot+
  AIS_is_improved_net_grob+
  inset_element(legend_AIS_cat, left=0.1, bottom = -0.1, 
                                   right = 0.3, top=0.4, ignore_tag = T)+
  AIS_is_improved_net_con_grob+inset_element(legend.AIS, left=-0.2, bottom = -0.1, 
                                   right = 0.3, top=0.4, ignore_tag = T)+
     AIS_is_improved.gcon$plot+theme(legend.position = "none")+
  wrap_elements(agg_MAP_net_plot,clip = F)+
  wrap_elements(agg_HR_net_plot, clip=F)

#Figure layout
layout <- c(
  # line 1
  area(t=1, l=1, r=6, b=2),
  # line 2
  area(t=3, l=1, r=2, b=4),area(t=3, l=3, r=4, b=4),area(t=3, l=5, r=6, b=4),
  # line 3
  area(t=5, l=1, r=2, b=6),area(t=5, l=3, r=4, b=6),area(t=5, l=5, r=6, b=6),
  # line 4
  area(t=7, l=1, r=2, b=8),area(t=7, l=3, r=4, b=8),area(t=7, l=5, r=6, b=8),
  # line 5
  area(t=9, l=1, r = 3, b=10),area(t=9, l=4, r = 6, b=10)
)

#Add figure options
figure2<-figure+plot_layout(design = layout)+
  plot_annotation(tag_levels = 'a',theme=theme(text = element_text(size=13)))

## Save figure
ggsave(figure2,file="figure 2.png", width = 12, height = 15.2, dpi = "retina")

figure2
```

Back to top

### 5.5.3 Figure 2 supplement 1

```
## read dendogram for panel h
dendo <- readPNG('dendogram.png')
dendo_grob<-rasterGrob(dendo, interpolate = T,width = unit(1.2, "native"), 
                       height = unit(1, "native"))

## compose figure
figure2sup1<-(fig2_sup1_a+ggtitle("Data processing")|fig2_sup1_b)/
  (fig2_sup1_c+ggtitle("Optimization Isomap solution")|fig2_sup1_d|fig2_sup1_e)/
  (fig2_sup1_f+ggtitle("Walktrap solution")|fig2_sup1_g|dendo_grob)+
  plot_annotation(tag_levels = 'a')

## Save figure
ggsave(figure2sup1,file="figure2sup1.png", width = 10, height = 8)

figure2sup1
```

Back to top

### 5.5.4 Figure 2 supplement 2

```
#AIS dis A
AIS_disA_con_net <- readPNG('AIS_disA_con.png')
AIS_disA_con_net_grob<-wrap_elements(rasterGrob(AIS_disA_con_net, interpolate = T,
                                       width = unit(1, "native"), 
                       height = unit(1, "native")),clip = F)
#AIS dis B
AIS_disB_con_net <- readPNG('AIS_disB_con.png')
AIS_disB_con_net_grob<-wrap_elements(rasterGrob(AIS_disB_con_net, interpolate = T,
                                       width = unit(1, "native"), 
                       height = unit(1, "native")),clip = F)
#AIS dis C
AIS_disC_con_net <- readPNG('AIS_disC_con.png')
AIS_disC_con_net_grob<-wrap_elements(rasterGrob(AIS_disC_con_net, interpolate = T,
                                       width = unit(1, "native"), 
                       height = unit(1, "native")),clip = F)
#AIS dis D
AIS_disD_con_net <- readPNG('AIS_disD_con.png')
AIS_disD_con_net_grob<-wrap_elements(rasterGrob(AIS_disD_con_net, interpolate = T,
                                       width = unit(1, "native"), 
                       height = unit(1, "native")),clip = F)
#AIS dis E
AIS_disE_con_net <- readPNG('AIS_disE_con.png')
AIS_disE_con_net_grob<-wrap_elements(rasterGrob(AIS_disE_con_net, interpolate = T,
                                       width = unit(1, "native"), 
                       height = unit(1, "native")),clip = F)

## Compose figure
figure<-AIS_disA_con_net_grob+wrap_elements(AIS_disA_plot, ignore_tag = T)+
  AIS_disB_con_net_grob+wrap_elements(AIS_disB_plot, ignore_tag = T)+
  AIS_disC_con_net_grob+wrap_elements(AIS_disC_plot, ignore_tag = T)+
  AIS_disD_con_net_grob+wrap_elements(AIS_disD_plot, ignore_tag = T)+
  AIS_disE_con_net_grob+wrap_elements(AIS_disE_plot, ignore_tag = T)+
  wrap_elements(AIS_dis1$plot+labs(fill="AIS dis.")+
    guides(fill=guide_legend(ncol = 1,byrow=F))+
    theme(legend.position = "right", legend.title = element_text()))

## Figure layout
layout <- c(
  ## line 1
  area(t=1, l=1, r=2, b=2),area(t=1, l=3, r=4, b=2),area(t=1, l=5, r=6, b=2),
  area(t=1, l=7, r=8, b=2),
  ## line 2
  area(t=3, l=1, r=2, b=4),area(t=3, l=3, r=4, b=4),area(t=3, l=5, r=6, b=4),
  area(t=3, l=7, r=8, b=4),
  ## line 3
  area(t=5, l=1, r=2, b=6),area(t=5, l=3, r = 4, b=6),area(t=5, l=5, r = 8, b=6)
)

## Add figure options
figure2sup2<-figure+plot_layout(design = layout)+
  plot_annotation(tag_levels = 'a')

## Save figure
ggsave(figure2sup2,file="figure2sup2.png", width = 12, height = 8, dpi = "retina")

figure2sup2
```

Back to top

# 6 MAP has a non-linear association with probability of recovery

## 6.1 Logistic regression

We build several models and compare them by Likelihood ratio test and cross validation errors. See methods in the paper for considered models.

```
## build dataframe for regression analysis
regression.df<-raw.df[!is.na(raw.df$AIS_is_improved),]
regression.df$AIS_is_improved<-as.character(regression.df$AIS_is_improved)

## specify outcome metric as 0: NO and 1: YES
regression.df$AIS_is_improved[regression.df$AIS_is_improved=='NO']<-0
regression.df$AIS_is_improved[regression.df$AIS_is_improved=='YES']<-1

regression.df$AIS_is_improved<-as.numeric(regression.df$AIS_is_improved)
regression.df$days_post_surgery_dish<-as.numeric(as.character(regression.df$days_post_surgery_dish))

## Fit the models
fit0<-glm(AIS_is_improved~1, data=regression.df, family = 'binomial', na.action = 'na.exclude')
fit1<-glm(AIS_is_improved~poly(mean.map,1), data=regression.df, family = 'binomial', na.action = 'na.exclude')
fit2<-glm(AIS_is_improved~poly(mean.map,2), data=regression.df, family = 'binomial', na.action = 'na.exclude')
fit3<-glm(AIS_is_improved~poly(mean.map,3), data=regression.df, family = 'binomial', na.action = 'na.exclude')
fit.ns2<-glm(AIS_is_improved~splines::ns(mean.map,2), data=regression.df, family = 'binomial', na.action = 'na.exclude')
fit.ns3<-glm(AIS_is_improved~splines::ns(mean.map,3), data=regression.df, family = 'binomial', na.action = 'na.exclude')
```

Back to top

### 6.1.1 Likelihood ratio test

```
anova(fit0, fit1, fit2, fit3, test ='LRT') # naive, linear and polynomials
```

```
## Analysis of Deviance Table
## 
## Model 1: AIS_is_improved ~ 1
## Model 2: AIS_is_improved ~ poly(mean.map, 1)
## Model 3: AIS_is_improved ~ poly(mean.map, 2)
## Model 4: AIS_is_improved ~ poly(mean.map, 3)
##   Resid. Df Resid. Dev Df Deviance Pr(>Chi)   
## 1       102     139.26                        
## 2       101     130.80  1   8.4672 0.003616 **
## 3       100     122.48  1   8.3202 0.003921 **
## 4        99     118.97  1   3.5022 0.061289 . 
## ---
## Signif. codes:  0 '***' 0.001 '**' 0.01 '*' 0.05 '.' 0.1 ' ' 1
```

```
anova(fit1, fit.ns2, test='LRT') # linear vs. n-spline 2
```

```
## Analysis of Deviance Table
## 
## Model 1: AIS_is_improved ~ poly(mean.map, 1)
## Model 2: AIS_is_improved ~ splines::ns(mean.map, 2)
##   Resid. Df Resid. Dev Df Deviance Pr(>Chi)   
## 1       101     130.80                        
## 2       100     122.29  1   8.5036 0.003544 **
## ---
## Signif. codes:  0 '***' 0.001 '**' 0.01 '*' 0.05 '.' 0.1 ' ' 1
```

```
anova(fit2,fit.ns3, test='LRT') # quadratic vs. n-spline 3
```

```
## Analysis of Deviance Table
## 
## Model 1: AIS_is_improved ~ poly(mean.map, 2)
## Model 2: AIS_is_improved ~ splines::ns(mean.map, 3)
##   Resid. Df Resid. Dev Df Deviance Pr(>Chi)  
## 1       100     122.48                       
## 2        99     119.13  1   3.3441  0.06745 .
## ---
## Signif. codes:  0 '***' 0.001 '**' 0.01 '*' 0.05 '.' 0.1 ' ' 1
```

### 6.1.2 LOOCV cross validation

Leave-one-out cross validation

```
set.seed(666)
cv.fit0<-cv.glm(regression.df, fit0) #the default K-CV is the number of observations, which means leave-one-out CV
cv.fit1<-cv.glm(regression.df, fit1)
cv.fit2<-cv.glm(regression.df, fit2)
cv.fit3<-cv.glm(regression.df, fit3)
cv.fit.ns2<-cv.glm(regression.df, fit.ns2)
cv.fit.ns3<-cv.glm(regression.df, fit.ns3)

CVerror0<-cv.fit0$delta[1] #Cross validation error
CVerror1<-cv.fit1$delta[1]#Cross validation error
CVerror2<-cv.fit2$delta[1]#Cross validation error
CVerror3<-cv.fit3$delta[1]#Cross validation error
CVerrorns2<-cv.fit.ns2$delta[1]#Cross validation error
CVerrorns3<-cv.fit.ns3$delta[1]#Cross validation error

model_names<-c('naive','linear','quadratic','cubic','n splin 2','n splin 3')
poly_cv_df<-data.frame(model=factor(model_names, levels=model_names),
                       CVerror=c(CVerror0,CVerror1,CVerror2,CVerror3,CVerrorns2,CVerrorns3))

poly_cv_df
```

```
##       model   CVerror
## 1     naive 0.2462514
## 2    linear 0.2319865
## 3 quadratic 0.2106499
## 4     cubic 0.2134627
## 5 n splin 2 0.2102387
## 6 n splin 3 0.2138096
```

Back to top

# 7 Factors influencing MAP association with recovery

## 7.1 FULL model LOOCV

Fitting the full model with LOOCV

```
## process the data for regression
regression.df$AIS_ad<-factor(regression.df$AIS_ad,
                             levels=c("A","B","C","D","E"))
regression.df$AIS_ad<-relevel(regression.df$AIS_ad, "E")

regression.df.f<-regression.df%>%select(mean.map, mean.HR, length_surg, days_post_surgery_dish, Age, AIS_ad,AIS_is_improved, Injury_level)

## format outcome variable as factor
regression.df.f$AIS_is_improved<-as.factor(regression.df.f$AIS_is_improved)

regression.df.f<-regression.df.f%>%
  mutate(AIS_is_improved_c = ifelse(AIS_is_improved==0,"NO","YES"))

## fit full model with LOOCV
ctrl <- trainControl(method = "LOOCV",savePredictions = "all",classProbs = T,
                     number = 1)

mod_fit <- train(AIS_is_improved_c ~ poly(mean.map,2)+mean.HR+length_surg+days_post_surgery_dish+Age+AIS_ad,
                 data=regression.df.f, method="glm", family="binomial",
                 trControl = ctrl,na.action = 'na.omit')

#Prediction probability
prob_predict_improve<-mod_fit$pred[,4]

summary(mod_fit)
```

```
## 
## Call:
## NULL
## 
## Deviance Residuals: 
##     Min       1Q   Median       3Q      Max  
## -1.7764  -0.8948  -0.3268   0.9220   2.7843  
## 
## Coefficients:
##                          Estimate Std. Error z value Pr(>|z|)  
## (Intercept)            -1.525e+01  1.218e+03  -0.013   0.9900  
## `poly(mean.map, 2)1`    7.650e+00  3.196e+00   2.394   0.0167 *
## `poly(mean.map, 2)2`   -8.179e+00  3.586e+00  -2.281   0.0225 *
## mean.HR                -2.087e-02  2.453e-02  -0.851   0.3949  
## length_surg             1.106e-03  1.520e-03   0.728   0.4669  
## days_post_surgery_dish  3.786e-03  1.099e-02   0.344   0.7306  
## Age                     8.243e-03  1.300e-02   0.634   0.5261  
## AIS_adA                 1.527e+01  1.218e+03   0.013   0.9900  
## AIS_adB                 1.585e+01  1.218e+03   0.013   0.9896  
## AIS_adC                 1.645e+01  1.218e+03   0.014   0.9892  
## AIS_adD                 1.454e+01  1.218e+03   0.012   0.9905  
## ---
## Signif. codes:  0 '***' 0.001 '**' 0.01 '*' 0.05 '.' 0.1 ' ' 1
## 
## (Dispersion parameter for binomial family taken to be 1)
## 
##     Null deviance: 125.800  on 92  degrees of freedom
## Residual deviance:  98.985  on 82  degrees of freedom
## AIC: 120.98
## 
## Number of Fisher Scoring iterations: 15
```

Back to top

## 7.2 Only poly MAP LOOCV

Fitting the model that only contains MAP (no covariates)

```
mod_fit_map <- train(AIS_is_improved_c ~ poly(mean.map,2),
                 data=regression.df.f, method="glm", family="binomial",
                 trControl = ctrl,na.action = 'na.omit')

#Prediction probability
prob_predict_improve_map<-mod_fit_map$pred[,4]

summary(mod_fit_map)
```

```
## 
## Call:
## NULL
## 
## Deviance Residuals: 
##     Min       1Q   Median       3Q      Max  
## -1.3164  -1.0195  -0.4705   1.0692   2.6590  
## 
## Coefficients:
##                      Estimate Std. Error z value Pr(>|z|)   
## (Intercept)           -0.5554     0.2422  -2.293  0.02183 * 
## `poly(mean.map, 2)1`   8.6287     2.9443   2.931  0.00338 **
## `poly(mean.map, 2)2`  -7.6019     3.0395  -2.501  0.01238 * 
## ---
## Signif. codes:  0 '***' 0.001 '**' 0.01 '*' 0.05 '.' 0.1 ' ' 1
## 
## (Dispersion parameter for binomial family taken to be 1)
## 
##     Null deviance: 139.26  on 102  degrees of freedom
## Residual deviance: 122.48  on 100  degrees of freedom
## AIC: 128.48
## 
## Number of Fisher Scoring iterations: 5
```

Back to top

## 7.3 Covariates only

Fitting the covariate only model

```
mod_fit_cov <- train(AIS_is_improved_c ~mean.HR+length_surg+days_post_surgery_dish+Age+AIS_ad,
                 data=regression.df.f, method="glm", family="binomial",
                 trControl = ctrl,na.action = 'na.omit')

#Prediction probability
prob_predict_improve_cov<-mod_fit_cov$pred[,4]

summary(mod_fit_cov)
```

```
## 
## Call:
## NULL
## 
## Deviance Residuals: 
##     Min       1Q   Median       3Q      Max  
## -1.8195  -0.9436  -0.6616   1.0479   1.8595  
## 
## Coefficients:
##                          Estimate Std. Error z value Pr(>|z|)
## (Intercept)            -1.585e+01  1.382e+03  -0.011    0.991
## mean.HR                -2.090e-02  2.295e-02  -0.911    0.362
## length_surg             1.631e-03  1.417e-03   1.151    0.250
## days_post_surgery_dish  1.055e-02  1.061e-02   0.993    0.320
## Age                     5.234e-03  1.235e-02   0.424    0.672
## AIS_adA                 1.574e+01  1.382e+03   0.011    0.991
## AIS_adB                 1.643e+01  1.382e+03   0.012    0.991
## AIS_adC                 1.715e+01  1.382e+03   0.012    0.990
## AIS_adD                 1.511e+01  1.382e+03   0.011    0.991
## 
## (Dispersion parameter for binomial family taken to be 1)
## 
##     Null deviance: 125.80  on 92  degrees of freedom
## Residual deviance: 110.54  on 84  degrees of freedom
## AIC: 128.54
## 
## Number of Fisher Scoring iterations: 15
```

Back to top

### 7.3.1 Figure 3

**Plot LOOCV estimated error all models and polynomial**

```
## LOOCV model plot
CV_models_plot<-ggplot(poly_cv_df, aes(model, CVerror))+
  geom_point()+
  geom_line(aes(as.numeric(model)))+
  theme_minimal()+
  ylab('Estimated LOOCV error')

## Plot polynomial

poly_plot<-regression.df%>%
  ggplot(aes(mean.map, AIS_is_improved))+
  geom_point(shape=16, color='black', alpha=0.2)+
  geom_smooth(method = "glm",method.args = list(family = "binomial"),
              se = T, formula = y~poly(x,2))+
  xlab('Mean MAP during surgery (mmHg)')+ylab(expression('Pr('*Delta*'AIS grade > 0)'))+
  theme_minimal(base_size = 11)

## add options to the figure
figure3<-CV_models_plot/poly_plot+plot_annotation(tag_levels = "a")

## save figure
ggsave(figure3, file="figure3.png", width = 4, height = 6)

figure3
```

Back to top

## 7.4 Only AIS-A

Models fitted to the AIS-A subcohort

### 7.4.1 Full LOOCV model A

```
## process the data for regression
regression.df.fA<-regression.df.f%>%
  filter(AIS_ad=="A")

mod_fit_A_poly <- train(AIS_is_improved_c ~ poly(mean.map,2)+mean.HR+length_surg+days_post_surgery_dish+Age,
                 data=regression.df.fA, method="glm", family="binomial",
                 trControl = ctrl,na.action = 'na.omit')
summary(mod_fit_A_poly)
```

```
## 
## Call:
## NULL
## 
## Deviance Residuals: 
##     Min       1Q   Median       3Q      Max  
## -1.6362  -0.8443  -0.2610   0.9818   2.5733  
## 
## Coefficients:
##                         Estimate Std. Error z value Pr(>|z|)  
## (Intercept)            -0.931740   3.433654  -0.271   0.7861  
## `poly(mean.map, 2)1`   10.798235   5.014412   2.153   0.0313 *
## `poly(mean.map, 2)2`   -6.738540   4.591262  -1.468   0.1422  
## mean.HR                -0.016780   0.035850  -0.468   0.6397  
## length_surg             0.003946   0.002624   1.504   0.1325  
## days_post_surgery_dish  0.006741   0.014127   0.477   0.6332  
## Age                    -0.012286   0.020506  -0.599   0.5491  
## ---
## Signif. codes:  0 '***' 0.001 '**' 0.01 '*' 0.05 '.' 0.1 ' ' 1
## 
## (Dispersion parameter for binomial family taken to be 1)
## 
##     Null deviance: 60.603  on 45  degrees of freedom
## Residual deviance: 44.593  on 39  degrees of freedom
## AIC: 58.593
## 
## Number of Fisher Scoring iterations: 6
```

Back to top

## 7.5 NLI

Models fitted with the NLI split cohorts

### 7.5.1 Model LOOCV Cervical

```
## process the data for regression
regression.df.fCer<-regression.df.f%>%
  filter(Injury_level=="Cervical")

regression.df.fCer<-droplevels(regression.df.fCer)

mod_fit_Cer <- train(AIS_is_improved_c ~ poly(mean.map,2)+mean.HR+length_surg+
                     days_post_surgery_dish+Age+AIS_ad,
                 data=regression.df.fCer, method="glm", family="binomial",
                 trControl = ctrl,na.action = 'na.omit')
summary(mod_fit_Cer)
```

```
## 
## Call:
## NULL
## 
## Deviance Residuals: 
##     Min       1Q   Median       3Q      Max  
## -1.6495  -0.7446  -0.2150   0.8919   3.0820  
## 
## Coefficients:
##                         Estimate Std. Error z value Pr(>|z|)  
## (Intercept)             2.747604   3.018504   0.910    0.363  
## `poly(mean.map, 2)1`    7.594951   3.056278   2.485    0.013 *
## `poly(mean.map, 2)2`   -7.528584   3.358419  -2.242    0.025 *
## mean.HR                -0.055241   0.034364  -1.608    0.108  
## length_surg             0.001420   0.001973   0.720    0.472  
## days_post_surgery_dish  0.002211   0.012134   0.182    0.855  
## Age                     0.007971   0.016526   0.482    0.630  
## AIS_adB                 0.300964   0.870861   0.346    0.730  
## AIS_adC                 0.745429   0.805491   0.925    0.355  
## AIS_adD                -0.747055   0.889038  -0.840    0.401  
## ---
## Signif. codes:  0 '***' 0.001 '**' 0.01 '*' 0.05 '.' 0.1 ' ' 1
## 
## (Dispersion parameter for binomial family taken to be 1)
## 
##     Null deviance: 89.724  on 64  degrees of freedom
## Residual deviance: 68.410  on 55  degrees of freedom
## AIC: 88.41
## 
## Number of Fisher Scoring iterations: 5
```

### 7.5.2 Model LOOCV Other

```
## process the data for regression
regression.df.fOther<-regression.df.f%>%
  filter(Injury_level=="Other")

regression.df.fOther<-droplevels(regression.df.fOther)

mod_fit_Other <- train(AIS_is_improved_c ~ poly(mean.map,2)+mean.HR+length_surg+
                     days_post_surgery_dish+Age+AIS_ad,
                 data=regression.df.fOther, method="glm", family="binomial",
                 trControl = ctrl,na.action = 'na.omit')
summary(mod_fit_Other)
```

```
## 
## Call:
## NULL
## 
## Deviance Residuals: 
##      Min        1Q    Median        3Q       Max  
## -1.24345  -0.73016  -0.25062   0.00013   1.86543  
## 
## Coefficients:
##                          Estimate Std. Error z value Pr(>|z|)
## (Intercept)            -1.870e+01  3.524e+03  -0.005    0.996
## `poly(mean.map, 2)1`   -1.453e-01  4.923e+00  -0.030    0.976
## `poly(mean.map, 2)2`   -8.262e+00  7.831e+00  -1.055    0.291
## mean.HR                 2.785e-04  6.493e-02   0.004    0.997
## length_surg             1.822e-03  5.428e-03   0.336    0.737
## days_post_surgery_dish  7.608e-02  6.136e-02   1.240    0.215
## Age                    -4.747e-02  5.156e-02  -0.921    0.357
## AIS_adA                 1.686e+01  3.524e+03   0.005    0.996
## AIS_adB                 1.738e+01  3.524e+03   0.005    0.996
## AIS_adC                 3.557e+01  5.782e+03   0.006    0.995
## AIS_adD                 1.727e+01  3.524e+03   0.005    0.996
## 
## (Dispersion parameter for binomial family taken to be 1)
## 
##     Null deviance: 33.503  on 27  degrees of freedom
## Residual deviance: 21.480  on 17  degrees of freedom
## AIC: 43.48
## 
## Number of Fisher Scoring iterations: 17
```

Back to top

## 7.6 Days to discharge

### 7.6.1 AIS improvement by days to discharge

```
summary(glm(AIS_is_improved ~ days_post_surgery_dish,
                 data=regression.df.f,family="binomial"))
```

```
## 
## Call:
## glm(formula = AIS_is_improved ~ days_post_surgery_dish, family = "binomial", 
##     data = regression.df.f)
## 
## Deviance Residuals: 
##     Min       1Q   Median       3Q      Max  
## -1.4692  -1.0089  -0.9717   1.3348   1.4024  
## 
## Coefficients:
##                         Estimate Std. Error z value Pr(>|z|)  
## (Intercept)            -0.552819   0.288248  -1.918   0.0551 .
## days_post_surgery_dish  0.009506   0.009602   0.990   0.3222  
## ---
## Signif. codes:  0 '***' 0.001 '**' 0.01 '*' 0.05 '.' 0.1 ' ' 1
## 
## (Dispersion parameter for binomial family taken to be 1)
## 
##     Null deviance: 131.48  on 96  degrees of freedom
## Residual deviance: 130.46  on 95  degrees of freedom
##   (6 observations deleted due to missingness)
## AIC: 134.46
## 
## Number of Fisher Scoring iterations: 4
```

#### 7.6.1.1 Interaction with MAP

```
summary(glm(AIS_is_improved ~ days_post_surgery_dish*poly(mean.map, 2),
                 data=regression.df.f,family="binomial"))
```

```
## 
## Call:
## glm(formula = AIS_is_improved ~ days_post_surgery_dish * poly(mean.map, 
##     2), family = "binomial", data = regression.df.f)
## 
## Deviance Residuals: 
##     Min       1Q   Median       3Q      Max  
## -1.4431  -1.0831  -0.4465   1.0762   2.6715  
## 
## Coefficients:
##                                            Estimate Std. Error z value Pr(>|z|)
## (Intercept)                               -0.560178   0.424271  -1.320   0.1867
## days_post_surgery_dish                     0.001422   0.022839   0.062   0.9503
## poly(mean.map, 2)1                        10.164340   4.995457   2.035   0.0419
## poly(mean.map, 2)2                        -7.412283   4.958915  -1.495   0.1350
## days_post_surgery_dish:poly(mean.map, 2)1 -0.117026   0.232813  -0.503   0.6152
## days_post_surgery_dish:poly(mean.map, 2)2 -0.033825   0.321774  -0.105   0.9163
##                                            
## (Intercept)                                
## days_post_surgery_dish                     
## poly(mean.map, 2)1                        *
## poly(mean.map, 2)2                         
## days_post_surgery_dish:poly(mean.map, 2)1  
## days_post_surgery_dish:poly(mean.map, 2)2  
## ---
## Signif. codes:  0 '***' 0.001 '**' 0.01 '*' 0.05 '.' 0.1 ' ' 1
## 
## (Dispersion parameter for binomial family taken to be 1)
## 
##     Null deviance: 131.48  on 96  degrees of freedom
## Residual deviance: 115.18  on 91  degrees of freedom
##   (6 observations deleted due to missingness)
## AIC: 127.18
## 
## Number of Fisher Scoring iterations: 5
```

### 7.6.2 Days to discharge by quadratic aMAP

```
summary(lm(days_post_surgery_dish ~ poly(mean.map, 2), data = regression.df.f))
```

```
## 
## Call:
## lm(formula = days_post_surgery_dish ~ poly(mean.map, 2), data = regression.df.f)
## 
## Residuals:
##     Min      1Q  Median      3Q     Max 
## -22.370 -13.137  -5.267   6.034 104.002 
## 
## Coefficients:
##                    Estimate Std. Error t value Pr(>|t|)    
## (Intercept)          20.810      2.203   9.445 2.78e-15 ***
## poly(mean.map, 2)1    6.711     22.086   0.304   0.7619    
## poly(mean.map, 2)2  -43.877     21.820  -2.011   0.0472 *  
## ---
## Signif. codes:  0 '***' 0.001 '**' 0.01 '*' 0.05 '.' 0.1 ' ' 1
## 
## Residual standard error: 21.7 on 94 degrees of freedom
##   (6 observations deleted due to missingness)
## Multiple R-squared:  0.04208,    Adjusted R-squared:  0.0217 
## F-statistic: 2.065 on 2 and 94 DF,  p-value: 0.1326
```

### 7.6.3 Full model - days to discharge

```
regression.df.fnoDays<-regression.df.f%>%
  select(AIS_is_improved_c, mean.map, mean.HR, length_surg, Age, AIS_ad, days_post_surgery_dish)%>%
  .[complete.cases(.),]

full_model<-train(AIS_is_improved_c ~ poly(mean.map,2)+mean.HR+days_post_surgery_dish+
                    length_surg+Age+AIS_ad,
                 data=regression.df.fnoDays, method="glm", family="binomial",
                 trControl = ctrl,na.action = 'na.omit')

mod_fit_noDays <- train(AIS_is_improved_c ~ poly(mean.map,2)+mean.HR+length_surg+Age+AIS_ad,
                 data=regression.df.fnoDays, method="glm", family="binomial",
                 trControl = ctrl,na.action = 'na.omit')

## Likelihood ratio test
anova(full_model$finalModel, mod_fit_noDays$finalModel, test = "LRT")
```

```
## Analysis of Deviance Table
## 
## Model 1: .outcome ~ `poly(mean.map, 2)1` + `poly(mean.map, 2)2` + mean.HR + 
##     days_post_surgery_dish + length_surg + Age + AIS_adA + AIS_adB + 
##     AIS_adC + AIS_adD
## Model 2: .outcome ~ `poly(mean.map, 2)1` + `poly(mean.map, 2)2` + mean.HR + 
##     length_surg + Age + AIS_adA + AIS_adB + AIS_adC + AIS_adD
##   Resid. Df Resid. Dev Df Deviance Pr(>Chi)
## 1        82     98.985                     
## 2        83     99.104 -1 -0.11943   0.7297
```

```
summary(full_model)
```

```
## 
## Call:
## NULL
## 
## Deviance Residuals: 
##     Min       1Q   Median       3Q      Max  
## -1.7764  -0.8948  -0.3268   0.9220   2.7843  
## 
## Coefficients:
##                          Estimate Std. Error z value Pr(>|z|)  
## (Intercept)            -1.530e+01  1.218e+03  -0.013   0.9900  
## `poly(mean.map, 2)1`    7.398e+00  3.112e+00   2.377   0.0174 *
## `poly(mean.map, 2)2`   -8.053e+00  3.530e+00  -2.281   0.0225 *
## mean.HR                -2.087e-02  2.453e-02  -0.851   0.3949  
## days_post_surgery_dish  3.786e-03  1.099e-02   0.344   0.7306  
## length_surg             1.106e-03  1.520e-03   0.728   0.4669  
## Age                     8.243e-03  1.300e-02   0.634   0.5261  
## AIS_adA                 1.527e+01  1.218e+03   0.013   0.9900  
## AIS_adB                 1.585e+01  1.218e+03   0.013   0.9896  
## AIS_adC                 1.645e+01  1.218e+03   0.014   0.9892  
## AIS_adD                 1.454e+01  1.218e+03   0.012   0.9905  
## ---
## Signif. codes:  0 '***' 0.001 '**' 0.01 '*' 0.05 '.' 0.1 ' ' 1
## 
## (Dispersion parameter for binomial family taken to be 1)
## 
##     Null deviance: 125.800  on 92  degrees of freedom
## Residual deviance:  98.985  on 82  degrees of freedom
## AIC: 120.98
## 
## Number of Fisher Scoring iterations: 15
```

```
summary(mod_fit_noDays)
```

```
## 
## Call:
## NULL
## 
## Deviance Residuals: 
##     Min       1Q   Median       3Q      Max  
## -1.7687  -0.9138  -0.3232   0.9264   2.8016  
## 
## Coefficients:
##                        Estimate Std. Error z value Pr(>|z|)  
## (Intercept)          -1.519e+01  1.206e+03  -0.013   0.9900  
## `poly(mean.map, 2)1`  7.445e+00  3.109e+00   2.395   0.0166 *
## `poly(mean.map, 2)2` -8.325e+00  3.465e+00  -2.403   0.0163 *
## mean.HR              -2.176e-02  2.441e-02  -0.892   0.3726  
## length_surg           1.070e-03  1.516e-03   0.706   0.4804  
## Age                   8.191e-03  1.300e-02   0.630   0.5287  
## AIS_adA               1.533e+01  1.206e+03   0.013   0.9899  
## AIS_adB               1.589e+01  1.206e+03   0.013   0.9895  
## AIS_adC               1.647e+01  1.206e+03   0.014   0.9891  
## AIS_adD               1.456e+01  1.206e+03   0.012   0.9904  
## ---
## Signif. codes:  0 '***' 0.001 '**' 0.01 '*' 0.05 '.' 0.1 ' ' 1
## 
## (Dispersion parameter for binomial family taken to be 1)
## 
##     Null deviance: 125.800  on 92  degrees of freedom
## Residual deviance:  99.104  on 83  degrees of freedom
## AIC: 119.1
## 
## Number of Fisher Scoring iterations: 15
```

# 8 Intraoperative MAP range from 76-[104-117] mmHg predicts recovery

The following section of the code works the MAP threshold determination as in the paper

## 8.1 Symetric threshold

### 8.1.1 Generate moving window vars

This section generates variables of the time inside/outside a increasing window centered on the mean MAP with higher prob. of improvement (90). It increments the window by 1mmHg each side at the time, so the first window is 89-91, second would be 88-92 and so on.

```
temp.list<-list()

## Calculate time out of MAP range for each range
for (t in 1:30){
  temp.df<-map_df_long%>%
    filter(!is.na(value))%>%
    group_by(Subject_ID)%>%
    summarise(num=sum(between(value, -Inf,90-t),between(value, 90+t,Inf)), time=num*5)
  colnames(temp.df)<-c('Subject_ID', 
                       paste('q5_MAP_out_',t, sep=''),
                       paste('Range ',90-t,'-',90+t, sep=''))
  temp.list[[t]]<-temp.df
}

## build a single dataframe for analysis
OR_MAP_threshold_out<-Reduce(function(x, y) merge(x, y, by='Subject_ID'), temp.list)
OR_MAP_threshold_out$AIS_is_improved<-raw.df$AIS_is_improved
OR_MAP_threshold_out_long<-OR_MAP_threshold_out%>%
  pivot_longer(-c(Subject_ID,AIS_is_improved))

# plot for window range schema
schema.plot.df<-map_df_long%>%filter(Subject_ID=='TSretro110')
schema.plot.df$n_q5<-1:dim(schema.plot.df)[1]

middle<-90
mw_symetric_plot<-ggplot(schema.plot.df, aes(n_q5, value))+
  geom_line()+
  coord_cartesian(xlim = c(0,120), ylim=c(69,115))+
  theme_minimal()+
  xlab('Time bin (Q5)')+ylab('MAP (mmHg)')+
  annotate("rect", xmin=85, xmax=130, ymin=65, ymax=115, fill="white")+
  annotate("rect", xmin=90, xmax=120, ymin=75, ymax=105, fill="grey85")+
  annotate("polygon", x=c(90, 120, 120, 90),y=c(89, 75, 105, 91), fill="white")+ 
  geom_hline(yintercept = middle, linetype='dashed')+
  annotate("segment", x=0, xend=90, y=middle+1, yend=middle+1, color="orange")+
  annotate("segment", x=0, xend=90, y=middle-1, yend=middle-1, color="orange")+
  annotate("segment", x=0, xend=100, y=middle+5, yend=middle+5, color="red")+
  annotate("segment", x=0, xend=100, y=middle-5, yend=middle-5, color="red")+
  annotate("segment", x=0, xend=110, y=middle+10, yend=middle+10, color="purple")+
  annotate("segment", x=0, xend=110, y=middle-10, yend=middle-10, color="purple")+
  annotate("segment", x=90, xend=90, y=89, yend=91, color="orange")+
  annotate("segment", x=100, xend=100, y=85, yend=95, color="red")+
  annotate("segment", x=110, xend=110, y=80, yend=100, color="purple")+
  annotate("segment", x=120, xend=120, y=75, yend=105, 
           color="black", linetype="dashed", 
           arrow=arrow(ends = "both",type = "closed", length = unit(2,"mm")))+
  annotate("text", x=105, y=110, label="Increase\n1 mmHg", size=4)+
  annotate("text", x=90, y=72, label="Time\noutside range", size=3, hjust=0)+
  annotate("rect",xmin=105, xmax=110, ymin=72, ymax=74, fill="grey85")+
  scale_x_continuous(breaks = seq(0,70,20))

mw_symetric_plot
```

Back to top

### 8.1.2 Assortativity of the range

Here we measure the assortativity of the network for the time outside each range

```
A_time_MAP_range<-data.frame(variable='', A=NA)
vars<-colnames(OR_MAP_threshold_out)[
  grepl(x = colnames(OR_MAP_threshold_out),pattern = 'Range')]

for(i in 1:length(vars)){
  temp<-assortativity(g,OR_MAP_threshold_out[,vars[i]],directed=F)
  temp.df<-data.frame(variable=vars[i],A=temp)
  A_time_MAP_range<-rbind(A_time_MAP_range, temp.df)
}

A_time_MAP_range<-A_time_MAP_range[-1,]
A_time_MAP_range$variable_num<-as.numeric(as.factor(A_time_MAP_range$variable))
A_time_MAP_range$variable<-str_replace(A_time_MAP_range$variable, 
                                       'Range ','')

labels_range<-A_time_MAP_range$variable
labels_range[seq(2,length(labels_range),2)]<-''
```

Back to top

### 8.1.3 Plot assortativity coeff

```
ac_range_plot<-ggplot(A_time_MAP_range, aes(variable_num, rev(A)))+
  geom_point()+
  scale_x_continuous(breaks =rev(A_time_MAP_range$variable_num),
                     labels = labels_range)+
  geom_vline(xintercept = 17, linetype='dashed')+
  theme_minimal()+
  geom_smooth(se=F, color='red', span=0.4, size=0.5)+
  ylab('Assortativity coeff. (Ar)')+xlab('MAP range (mmHg)')+
  theme(axis.text.x = element_text(angle = 90, 
                                   hjust = 0.5, vjust = 0.5, size=9),
        axis.ticks.x = element_line(color='black'))

# ggsave('Ar range.png', width = 3, height = 2.5, dpi='print')
ac_range_plot
```

Network for range 73-107 found by assortativity analysis

```
mapping$range_73_107<-OR_MAP_threshold_out$`Range 73-107`
range_73_107.net<-plot_net('range_73_107','mean','xx', ranges=c(1,30),
                           network_name = "range_73_107_net")

agg_plot_df$range_73_107<-range_73_107.net$agg_values

agg_plot_73_107<-ggplot(agg_plot_df, aes(range_73_107, AIS, fill=range_73_107))+
  geom_point(shape=21, size=2)+
  scale_fill_gradientn(colors=net.color)+
  xlab('Mean cluster time out of MAP\nrange 73-107 mmHg')+
  ylab('Proportion of\n AIS improve.')+labs(fill='Mean\ntime out')+
  theme_minimal(base_size = 11)+
  geom_smooth(span=1, se=F)

agg_plot_73_107
```

### 8.1.4 LASSO

logistic regression with LASSO regularization and cross-validation to find the range of MAP that is most predictive of AIS improvement.

#### 8.1.4.1 Data processing for LASSO

```
lasso.df<-OR_MAP_threshold_out%>%
  filter(!is.na(AIS_is_improved))

vars<-colnames(lasso.df)[grepl(x = colnames(lasso.df), 
                               pattern = 'Range')]
lasso.x<-as.matrix(lasso.df%>%dplyr::select(vars))
```

#### 8.1.4.2 Fit logistic LASSO regression

Lambda is determined by cross validation. N folds controls for the number of folds CV. Setting nfolds for 103 (number of observations) implies doing LOOCV for lambda (see glmnet and glmnetUtils packAges for details)

```
logLasso<-cv.glmnet(x=lasso.x, y=lasso.df$AIS_is_improved,family='binomial',
                    alpha=1,nfolds=103)
#Coefficients
coef(logLasso$glmnet.fit,s=logLasso$lambda.1se)
```

```
## 31 x 1 sparse Matrix of class "dgCMatrix"
##                        s1
## (Intercept)  -0.176419664
## Range 89-91   .          
## Range 88-92   .          
## Range 87-93   .          
## Range 86-94   .          
## Range 85-95   .          
## Range 84-96   .          
## Range 83-97   .          
## Range 82-98   .          
## Range 81-99   .          
## Range 80-100  .          
## Range 79-101  .          
## Range 78-102  .          
## Range 77-103  .          
## Range 76-104 -0.001645099
## Range 75-105  .          
## Range 74-106  .          
## Range 73-107  .          
## Range 72-108  .          
## Range 71-109  .          
## Range 70-110  .          
## Range 69-111  .          
## Range 68-112  .          
## Range 67-113  .          
## Range 66-114  .          
## Range 65-115  .          
## Range 64-116  .          
## Range 63-117  .          
## Range 62-118  .          
## Range 61-119  .          
## Range 60-120  .
```

#### 8.1.4.3 LASSO range cross validation

Logistic cross LASSO range solution validation

```
log.CV.df<-OR_MAP_threshold_out%>%
  select(`Range 76-104`,AIS_is_improved)%>%
  filter(!is.na(AIS_is_improved))

ctrl <- trainControl(method = "LOOCV",savePredictions = TRUE, classProbs = T)

mod_fit_sym <- train(AIS_is_improved ~ `Range 76-104`,log.CV.df, method="glm", family="binomial",trControl = ctrl)

summary(mod_fit_sym)
```

```
## 
## Call:
## NULL
## 
## Deviance Residuals: 
##     Min       1Q   Median       3Q      Max  
## -1.3225  -1.0264  -0.7148   1.1699   2.4187  
## 
## Coefficients:
##                       Estimate Std. Error z value Pr(>|z|)  
## (Intercept)           0.368281   0.333798   1.103   0.2699  
## `\\`Range 76-104\\`` -0.006677   0.002602  -2.566   0.0103 *
## ---
## Signif. codes:  0 '***' 0.001 '**' 0.01 '*' 0.05 '.' 0.1 ' ' 1
## 
## (Dispersion parameter for binomial family taken to be 1)
## 
##     Null deviance: 139.26  on 102  degrees of freedom
## Residual deviance: 130.50  on 101  degrees of freedom
## AIC: 134.5
## 
## Number of Fisher Scoring iterations: 4
```

```
mod_fit_sym
```

```
## Generalized Linear Model 
## 
## 103 samples
##   1 predictor
##   2 classes: 'NO', 'YES' 
## 
## No pre-processing
## Resampling: Leave-One-Out Cross-Validation 
## Summary of sample sizes: 102, 102, 102, 102, 102, 102, ... 
## Resampling results:
## 
##   Accuracy   Kappa    
##   0.6116505  0.1584967
```

## 8.2 Upper limit

### 8.2.1 Generate asymetric moving window

This section generates variables of the time inside/outside a increasing window fixed on the mean MAP 76 on the lower bound. It increments the window by 1mmHg each time, so the first window is 76-77, second would be 76-78 and so on.

```
temp.list<-list()
# map_df_long<-map.wide%>%pivot_longer(-Subject_ID,names_to = 'variable', values_to = 'value')

## calculate time outside MAP range for each range
for (t in 1:44){
  temp.df<-map_df_long%>%
    filter(!is.na(value))%>%
    group_by(Subject_ID)%>%
    summarise(num=sum(between(value, -Inf,76),between(value, 76+t,Inf)), time=num*5)
  colnames(temp.df)<-c('Subject_ID', 
                       paste('q5_MAP_out_',t, sep=''),
                       paste('Range ',76,'-',76+t, sep=''))
  temp.list[[t]]<-temp.df
}

## build single dataframe for analysis
OR_MAP_threshold_out_upper<-Reduce(function(x, y) merge(x, y, by='Subject_ID'), temp.list)
OR_MAP_threshold_out_upper$AIS_is_improved<-raw.df$AIS_is_improved
OR_MAP_threshold_out_upper_long<-OR_MAP_threshold_out_upper%>%
  pivot_longer(-c(Subject_ID,AIS_is_improved))

# plot for window range schema
schema.plot.df_upper<-map_df_long%>%filter(Subject_ID=='TSretro110')
schema.plot.df_upper$n_q5<-1:dim(schema.plot.df_upper)[1]

middle<-76
mw_asymetric_plot<-ggplot(schema.plot.df_upper, aes(n_q5, value))+
  geom_line()+
  coord_cartesian(xlim = c(0,120), ylim=c(69,115))+
  theme_minimal()+
  xlab('Time bin (Q5)')+ylab('MAP (mmHg)')+
  annotate("rect", xmin=85, xmax=130, ymin=76, ymax=115, fill="white")+
  annotate("rect", xmin=90, xmax=120, ymin=65, ymax=91, fill="grey85")+
  annotate("polygon", x=c(90, 120, 120, 90),y=c(76, 76, 91, 77), fill="white")+ 
  geom_hline(yintercept = middle, linetype='dashed')+
  annotate("segment", x=0, xend=90, y=middle+1, yend=middle+1, color="orange")+
  annotate("segment", x=0, xend=100, y=middle+5, yend=middle+5, color="red")+
  annotate("segment", x=0, xend=110, y=middle+10, yend=middle+10, color="purple")+
  annotate("segment", x=90, xend=90, y=76, yend=77, color="orange")+
  annotate("segment", x=100, xend=100, y=76, yend=81, color="red")+
  annotate("segment", x=110, xend=110, y=76, yend=86, color="purple")+
  annotate("segment", x=120, xend=120, y=76, yend=91, 
           color="black", linetype="dashed", 
           arrow=arrow(ends = "both",type = "closed", length = unit(2,"mm")))+
  annotate("text", x=105, y=96, label="Increase\n1 mmHg", size=4)+
  annotate("text", x=60, y=72, label="Time\noutside range", size=3, hjust=0)+
  annotate("rect",xmin=75, xmax=80, ymin=72, ymax=74, fill="grey85")+
  scale_x_continuous(breaks = seq(0,70,20))

mw_asymetric_plot
```

### 8.2.2 Assortativity of the range

```
A_time_MAP_range_upper<-data.frame(variable='', A=NA)
vars_upper<-colnames(OR_MAP_threshold_out_upper)[
  grepl(x = colnames(OR_MAP_threshold_out_upper),pattern = 'Range')]

for(i in 1:length(vars_upper)){
  temp<-assortativity(g,OR_MAP_threshold_out_upper[,vars_upper[i]],directed=F)
  temp.df<-data.frame(variable=vars_upper[i],A=temp)
  A_time_MAP_range_upper<-rbind(A_time_MAP_range_upper, temp.df)
}

A_time_MAP_range_upper<-A_time_MAP_range_upper[-1,]
A_time_MAP_range_upper$variable_num<-1:44
A_time_MAP_range_upper$variable<-str_replace(A_time_MAP_range_upper$variable, 
                                       'Range ','')

labels_range_upper<-A_time_MAP_range_upper$variable
labels_range_upper[seq(2,length(labels_range_upper),2)]<-''
```

### 8.2.3 Plot assortativity coeff

```
ac_range_plot_upper<-ggplot(A_time_MAP_range_upper, aes(variable_num, A))+
  geom_point()+
  scale_x_continuous(breaks =A_time_MAP_range_upper$variable_num,
                     labels = labels_range_upper)+
  geom_vline(xintercept = 40, linetype='dashed')+
  theme_minimal()+
  geom_smooth(se=F, color='red', span=0.4, size=0.5)+
  ylab('Assortativity coeff. (Ar)')+xlab('MAP range (mmHg)')+
  theme(axis.text.x = element_text(angle = 90, 
                                   hjust = 0.5, vjust = 0.5, size=9),
        axis.ticks.x = element_line(color='black'))

# ggsave('Ar range.png', width = 3, height = 2.5, dpi='print')
ac_range_plot_upper
```

Network for range 76-116 found by assortativity analysis

```
mapping$range_76_116<-OR_MAP_threshold_out_upper$`Range 76-116`
range_76_116.net<-plot_net('range_76_116','mean','xx', ranges=c(1,30),
                           network_name = "range_76_116_net")

agg_plot_df$range_76_116<-range_76_116.net$agg_values

agg_plot_76_116<-ggplot(agg_plot_df, aes(range_76_116, AIS, fill=range_76_116))+
  geom_point(shape=21, size=2)+
  scale_fill_gradientn(colors=net.color)+
  xlab('Mean cluster time out of MAP\nrange 76-116 mmHg')+
  ylab('Proportion of\n AIS improve.')+labs(fill='Mean\ntime out')+
  theme_minimal(base_size = 11)+
  geom_smooth(span=1, se=F)

agg_plot_76_116
```

### 8.2.4 LASSO upper limit

#### 8.2.4.1 Data processing for LASSO

```
lasso.df_upper<-OR_MAP_threshold_out_upper%>%
  filter(!is.na(AIS_is_improved))

vars_upper<-colnames(lasso.df_upper)[grepl(x = colnames(lasso.df_upper), 
                               pattern = 'Range')]
lasso.x_upper<-as.matrix(lasso.df_upper%>%dplyr::select(vars_upper))
```

#### 8.2.4.2 Fit logistic LASSO regression

Lambda is determined by cross validation. N folds controls for the number of folds CV. Setting nfolds for 103 (number of observations) implies doing LOOCV for lambda (see glmnet and glmnetUtils packAges for details)

```
logLasso_upper<-cv.glmnet(x=lasso.x_upper, y=lasso.df_upper$AIS_is_improved,family='binomial',
                    alpha=1,nfolds=103)

#Coefficients
coef(logLasso_upper$glmnet.fit,s=logLasso_upper$lambda.1se)
```

```
## 45 x 1 sparse Matrix of class "dgCMatrix"
##                         s1
## (Intercept)  -0.3251877484
## Range 76-77   .           
## Range 76-78   .           
## Range 76-79   .           
## Range 76-80   .           
## Range 76-81   .           
## Range 76-82   .           
## Range 76-83   .           
## Range 76-84   .           
## Range 76-85   .           
## Range 76-86   .           
## Range 76-87   .           
## Range 76-88   .           
## Range 76-89   .           
## Range 76-90   .           
## Range 76-91   .           
## Range 76-92   .           
## Range 76-93   .           
## Range 76-94   .           
## Range 76-95   .           
## Range 76-96   .           
## Range 76-97   .           
## Range 76-98   .           
## Range 76-99   .           
## Range 76-100  .           
## Range 76-101  .           
## Range 76-102  .           
## Range 76-103  .           
## Range 76-104  .           
## Range 76-105  .           
## Range 76-106  .           
## Range 76-107  .           
## Range 76-108  .           
## Range 76-109  .           
## Range 76-110  .           
## Range 76-111  .           
## Range 76-112  .           
## Range 76-113  .           
## Range 76-114  .           
## Range 76-115  .           
## Range 76-116  .           
## Range 76-117 -0.0004923292
## Range 76-118  .           
## Range 76-119  .           
## Range 76-120  .
```

#### 8.2.4.3 LASSO range cross validation

```
log.CV.df_upper<-OR_MAP_threshold_out_upper%>%
  select(`Range 76-117`,AIS_is_improved)%>%
  filter(!is.na(AIS_is_improved))

ctrl <- trainControl(method = "LOOCV",savePredictions = TRUE, classProbs = T)

mod_fit_asym <- train(AIS_is_improved ~ `Range 76-117`,log.CV.df_upper, method="glm", family="binomial",trControl = ctrl)

summary(mod_fit_asym)
```

```
## 
## Call:
## NULL
## 
## Deviance Residuals: 
##     Min       1Q   Median       3Q      Max  
## -1.3019  -1.1081  -0.6163   1.1391   2.6699  
## 
## Coefficients:
##                       Estimate Std. Error z value Pr(>|z|)   
## (Intercept)           0.288111   0.287654   1.002  0.31654   
## `\\`Range 76-117\\`` -0.007884   0.002788  -2.828  0.00468 **
## ---
## Signif. codes:  0 '***' 0.001 '**' 0.01 '*' 0.05 '.' 0.1 ' ' 1
## 
## (Dispersion parameter for binomial family taken to be 1)
## 
##     Null deviance: 139.26  on 102  degrees of freedom
## Residual deviance: 127.65  on 101  degrees of freedom
## AIC: 131.65
## 
## Number of Fisher Scoring iterations: 4
```

```
mod_fit_asym
```

```
## Generalized Linear Model 
## 
## 103 samples
##   1 predictor
##   2 classes: 'NO', 'YES' 
## 
## No pre-processing
## Resampling: Leave-One-Out Cross-Validation 
## Summary of sample sizes: 102, 102, 102, 102, 102, 102, ... 
## Resampling results:
## 
##   Accuracy   Kappa    
##   0.5728155  0.1022187
```

## 8.3 Figure 4

```
OR_MAP_threshold_out$AIS_is_improved<-as.character(OR_MAP_threshold_out$AIS_is_improved)
OR_MAP_threshold_out$AIS_is_improved[OR_MAP_threshold_out$AIS_is_improved=='NO']<-0
OR_MAP_threshold_out$AIS_is_improved[OR_MAP_threshold_out$AIS_is_improved=='YES']<-1
OR_MAP_threshold_out$AIS_is_improved<-as.numeric(OR_MAP_threshold_out$AIS_is_improved)

range_log_plot<-OR_MAP_threshold_out%>%filter(!is.na(AIS_is_improved))%>%
  select(`Range 76-104`,AIS_is_improved)%>%
  ggplot(aes(`Range 76-104`, AIS_is_improved))+
  geom_point(shape=16, color='black', alpha=0.2)+
  geom_smooth(method = "glm",method.args = list(family = "binomial"),
              se = T)+
  xlab('Estimated time outside\n 76-104mmHg MAP range (minutes)')+ylab(expression('Pr('*Delta*'AIS grade > 0)'))+
  annotate("text", label = "Time MAP outside\n76-104mmHg", x=300, y=0.8)+
  ggtitle("Confirmatory Logistic LASSO")+
  theme_minimal()

OR_MAP_threshold_out_upper$AIS_is_improved<-as.character(OR_MAP_threshold_out_upper$AIS_is_improved)
OR_MAP_threshold_out_upper$AIS_is_improved[OR_MAP_threshold_out_upper$AIS_is_improved=='NO']<-0
OR_MAP_threshold_out_upper$AIS_is_improved[OR_MAP_threshold_out_upper$AIS_is_improved=='YES']<-1
OR_MAP_threshold_out_upper$AIS_is_improved<-as.numeric(OR_MAP_threshold_out_upper$AIS_is_improved)

range_log_plot_upper<-OR_MAP_threshold_out_upper%>%filter(!is.na(AIS_is_improved))%>%
  select(`Range 76-117`,AIS_is_improved)%>%
  ggplot(aes(`Range 76-117`, AIS_is_improved))+
  geom_point(shape=16, color='black', alpha=0.2)+
  geom_smooth(method = "glm",method.args = list(family = "binomial"),
              se = T)+
  xlab('Estimated time outside\n76-117mmHg MAP range (minutes)')+ylab(expression('Pr('*Delta*'AIS grade > 0)'))+
  annotate("text", label = "Time MAP outside\n76-117mmHg", x=300, y=0.8)+
  ggtitle("Confirmatory Logistic LASSO")+
  theme_minimal()

figure4<-(mw_symetric_plot+ggtitle("Symmetric MAP threshold")|ac_range_plot+ggtitle("Exploratory network MAP range")|range_log_plot)/
  (mw_asymetric_plot+ggtitle("Upper limit threshold")|ac_range_plot_upper+ggtitle("Exploratory network MAP range")|range_log_plot_upper)

figure4<-figure4+plot_annotation(tag_levels = 'a')

ggsave(figure4,file="figure 4.png", width = 12, height = 7)

figure4
```

### 8.3.1 Fig4 supplement 1

```
fig4s1_a<-readPNG("range_73_107_net.png")
fig4s1_a<-wrap_elements(rasterGrob(fig4s1_a, interpolate = T,
                                       width = unit(1, "native"), 
                       height = unit(1, "native")))

fig4s1_b<-readPNG("range_73_107_net_con.png")
fig4s1_b<-wrap_elements(rasterGrob(fig4s1_b, interpolate = T,
                                       width = unit(1, "native"), 
                       height = unit(1, "native")))

fig4s1_d<-readPNG("range_76_116_net.png")
fig4s1_d<-wrap_elements(rasterGrob(fig4s1_d, interpolate = T,
                                       width = unit(1, "native"), 
                       height = unit(1, "native")))

fig4s1_e<-readPNG("range_76_116_net_con.png")
fig4s1_e<-wrap_elements(rasterGrob(fig4s1_e, interpolate = T,
                                       width = unit(1, "native"), 
                       height = unit(1, "native")))

fig4s1<-fig4s1_a+fig4s1_b+wrap_elements(agg_plot_73_107)+
           fig4s1_d+fig4s1_e+wrap_elements(agg_plot_76_116)

layout <- c(
  # line 1
  area(t=1, l=1, r=2, b=2),area(t=1, l=3, r=4, b=2),area(t=1, l=5, r=7, b=2),
  # line 2
  area(t=3, l=1, r=2, b=4),area(t=3, l=3, r=4, b=4),area(t=3, l=5, r=7, b=4)
)

fig4s1<-fig4s1+plot_layout(design = layout)+plot_annotation(tag_levels = 'a',theme = theme(text = element_text(size=13)))

ggsave(fig4s1,file="fig4 supplement 1.png", width = 12, height = 6)

fig4s1
```

### 8.3.2 Fig4 supplement 2

```
#Plot LASSO solutions
fig4s2_a<-rasterGrob({
  png(filename = "temp.png",res = 150)
    plot(logLasso)
  dev.off()
  readPNG("temp.png")
},interpolate = T,width = unit(1, "native"), height = unit(1, "native"))

fig4s2_b<-rasterGrob({
  png(filename = "temp.png",res = 150)
    plot(logLasso$glmnet.fit, xvar='lam',label = T)
  dev.off()
  readPNG("temp.png")
},interpolate = T,width = unit(1, "native"), height = unit(1, "native"))


# Plotting the individual panels
fig4s2_c<-OR_MAP_threshold_out%>%
  select(colnames(OR_MAP_threshold_out)[
    str_detect(colnames(OR_MAP_threshold_out),"Range")],
    AIS_is_improved)%>%
  pivot_longer(-AIS_is_improved)%>%
  mutate(name = factor(name, levels = unique(name)))%>%
  ggplot(aes(value, AIS_is_improved))+
    geom_point(shape=16, color='black', alpha=0.2)+
    geom_smooth(method = "glm",method.args = list(family = "binomial"),
              se = T)+
    xlab('Estimated time outside MAP')+
    ylab(expression('Pr('*Delta*'AIS grade > 0)'))+
  theme_minimal(base_size = 11)+
  facet_wrap(~name)

fig4s2<-wrap_elements(fig4s2_a)+wrap_elements(fig4s2_b)+fig4s2_c

layout <- c(
  # line 1
  area(t=1, l=1, r=2, b=2),area(t=1, l=3, r=4, b=2),
  # line 2
  area(t=3, l=1, r=4, b=6)
)

fig4s2<-fig4s2+plot_layout(design = layout)+plot_annotation(tag_levels = 'a',theme = theme(text = element_text(size=13)))

ggsave(fig4s2,file="fig4 supplement 2.png", width = 8, height = 10)

fig4s2
```

### 8.3.3 Fig4 supplement 3

```
#Plot LASSO solutions
fig4s3_a<-rasterGrob({
  png(filename = "temp.png",res = 150)
    plot(logLasso_upper)
  dev.off()
  readPNG("temp.png")
},interpolate = T,width = unit(1, "native"), height = unit(1, "native"))

fig4s3_b<-rasterGrob({
  png(filename = "temp.png",res = 150)
    plot(logLasso_upper$glmnet.fit, xvar='lam',label = T)
  dev.off()
  readPNG("temp.png")
},interpolate = T,width = unit(1, "native"), height = unit(1, "native"))


# Plotting the individual panels
fig4s3_c<-OR_MAP_threshold_out_upper%>%
  select(colnames(OR_MAP_threshold_out_upper)[
    str_detect(colnames(OR_MAP_threshold_out_upper),"Range")],
    AIS_is_improved)%>%
  pivot_longer(-AIS_is_improved)%>%
  mutate(name = factor(name, levels = unique(name)))%>%
  ggplot(aes(value, AIS_is_improved))+
    geom_point(shape=16, color='black', alpha=0.2)+
    geom_smooth(method = "glm",method.args = list(family = "binomial"),
              se = T)+
    xlab('Estimated time outside MAP')+
    ylab(expression('Pr('*Delta*'AIS grade > 0)'))+
  theme_minimal(base_size = 11)+
  facet_wrap(~name)

fig4s3<-wrap_elements(fig4s3_a)+wrap_elements(fig4s3_b)+fig4s3_c

layout <- c(
  # line 1
  area(t=1, l=1, r=2, b=2),area(t=1, l=3, r=4, b=2),
  # line 2
  area(t=3, l=1, r=4, b=6)
)

fig4s3<-fig4s3+plot_layout(design = layout)+plot_annotation(tag_levels = 'a',theme = theme(text = element_text(size=13)))

ggsave(fig4s3,file="fig4 supplement 3.png", width = 8, height = 10)

fig4s3
```

# 9 Prediction models

## 9.1 Predicting AIS improvement

### 9.1.1 Data preprocessing

Process the data for prediction model

```
## build dataframe for analysis
prediction.df<-regression.df%>%
  left_join(OR_MAP_threshold_out_upper[,c("Subject_ID","Range 76-117")])%>%
  left_join(OR_MAP_threshold_out[,c("Subject_ID","Range 76-104")])

prediction.df.f<-prediction.df%>%
  select(mean.map, mean.HR, length_surg, days_post_surgery_dish, Age,
         AIS_ad,AIS_is_improved, Injury_level, 
        `Range 76-117`,`Range 76-104`)%>%
  mutate(AIS_is_improved_c = ifelse(AIS_is_improved==0,"NO","YES"))%>%
  rename(range_76_104 = `Range 76-104`, range_76_117 = `Range 76-117`)%>%
  filter(complete.cases(.))
```

### 9.1.2 Search for best model

Search for the best model as for AIC. Single terms and pairwise interaction are allowed.

```
model_search<-glmulti(AIS_is_improved~poly(mean.map,2)+mean.HR+length_surg+ days_post_surgery_dish+Age+AIS_ad+Injury_level+range_76_104+range_76_117, 
                 data = prediction.df.f,family="binomial", level = 1, crit = "aicc", plotty = F, report = F)

## Extract the model that minimizes AIC
best_model<-model_search@objects[[1]]
```

### 9.1.3 Best model

```
best_model
```

```
## 
## Call:  fitfunc(formula = as.formula(x), family = "binomial", data = data)
## 
## Coefficients:
##        (Intercept)  poly(mean.map, 2)1  poly(mean.map, 2)2  
##            -0.5557              7.8695             -7.7678  
## 
## Degrees of Freedom: 92 Total (i.e. Null);  90 Residual
## Null Deviance:       125.8 
## Residual Deviance: 110.2     AIC: 116.2
```

### 9.1.4 Prediction models

Next we build a prediction model with the terms from the best model in the previous search.

```
## Best model
mod_fit_best <- train(AIS_is_improved_c~poly(mean.map,2)+AIS_ad, 
                 data = prediction.df.f, method="glm", family="binomial",
                 trControl = ctrl,na.action = 'na.omit')

#Prediction probability
prob_predict_improve_best<-mod_fit_best$pred[,4]

model_prediction_best<-prediction_fun(prob_predict_improve_best,
                        prediction.df.f$AIS_is_improved,ref = "1")


##No MAP
mod_fit_best_noMap <- train(AIS_is_improved_c~AIS_ad, 
                 data = prediction.df.f, method="glm", family="binomial",
                 trControl = ctrl,na.action = 'na.omit')

#Prediction probability
prob_predict_improve_best_noMAP<-mod_fit_best_noMap$pred[,4]

model_prediction_best_noMAP<-prediction_fun(prob_predict_improve_best_noMAP,
                        prediction.df.f$AIS_is_improved,ref = "1")

model_prediction_best$conf_mat
```

```
## Confusion Matrix and Statistics
## 
##           Reference
## Prediction  0  1
##          0 41 11
##          1 14 27
##                                           
##                Accuracy : 0.7312          
##                  95% CI : (0.6292, 0.8179)
##     No Information Rate : 0.5914          
##     P-Value [Acc > NIR] : 0.003538        
##                                           
##                   Kappa : 0.4505          
##                                           
##  Mcnemar's Test P-Value : 0.689157        
##                                           
##             Sensitivity : 0.7105          
##             Specificity : 0.7455          
##          Pos Pred Value : 0.6585          
##          Neg Pred Value : 0.7885          
##              Prevalence : 0.4086          
##          Detection Rate : 0.2903          
##    Detection Prevalence : 0.4409          
##       Balanced Accuracy : 0.7280          
##                                           
##        'Positive' Class : 1               
##
```

```
model_prediction_best_noMAP$conf_mat
```

```
## Confusion Matrix and Statistics
## 
##           Reference
## Prediction  0  1
##          0 29 21
##          1 26 17
##                                           
##                Accuracy : 0.4946          
##                  95% CI : (0.3893, 0.6003)
##     No Information Rate : 0.5914          
##     P-Value [Acc > NIR] : 0.9767          
##                                           
##                   Kappa : -0.0249         
##                                           
##  Mcnemar's Test P-Value : 0.5596          
##                                           
##             Sensitivity : 0.4474          
##             Specificity : 0.5273          
##          Pos Pred Value : 0.3953          
##          Neg Pred Value : 0.5800          
##              Prevalence : 0.4086          
##          Detection Rate : 0.1828          
##    Detection Prevalence : 0.4624          
##       Balanced Accuracy : 0.4873          
##                                           
##        'Positive' Class : 1               
##
```

```
coef(mod_fit_best$finalModel)
```

```
##          (Intercept) `poly(mean.map, 2)1` `poly(mean.map, 2)2` 
##           -16.240157             7.373723            -8.214947 
##              AIS_adA              AIS_adB              AIS_adC 
##            15.546328            16.181807            16.752314 
##              AIS_adD 
##            14.828155
```

## 9.2 Predicting AIS discharge

Process the data for prediction model

```
prediction.df.f_dis<-prediction.df%>%
  select(mean.map, mean.HR, length_surg, days_post_surgery_dish, Age,
         AIS_ad,AIS_is_improved, Injury_level, AIS_dis,
        `Range 76-117`,`Range 76-104`)%>%
  mutate(A_dis = ifelse(AIS_dis == "A", 1, 0),
         D_dis = ifelse(AIS_dis == "D", 1, 0),
         A_dis_f = ifelse(AIS_dis == "A", "A", "nonA"),
         D_dis_f = ifelse(AIS_dis == "D", "D", "nonD"))%>%
  rename(range_76_104 = `Range 76-104`, range_76_117 = `Range 76-117`)%>%
  filter(complete.cases(.))
```

### 9.2.1 Predicting A discharge

#### 9.2.1.1 Search for best model

Search for the best model as for AIC.

```
model_search_disA<-glmulti(A_dis~poly(mean.map,2)+mean.HR+length_surg+ days_post_surgery_dish+Age+AIS_ad+Injury_level+range_76_104+range_76_117, 
                 data = prediction.df.f_dis,family="binomial", level = 1,
                 crit = "aicc", plotty = F, report = F)

## Extract the model that minimizes AIC
best_model_disA<-model_search_disA@objects[[1]]
```

#### 9.2.1.2 Best model

```
best_model_disA
```

```
## 
## Call:  fitfunc(formula = as.formula(x), family = "binomial", data = data)
## 
## Coefficients:
##        (Intercept)             AIS_adA             AIS_adB             AIS_adC  
##          -20.46638            22.81462            20.38324            19.00705  
##            AIS_adD  poly(mean.map, 2)1  poly(mean.map, 2)2   Injury_levelOther  
##           -0.21798           -27.03167            17.13848             1.22894  
##       range_76_117  
##           -0.01778  
## 
## Degrees of Freedom: 92 Total (i.e. Null);  84 Residual
## Null Deviance:       119.7 
## Residual Deviance: 58    AIC: 76
```

#### 9.2.1.3 Prediction models

Next we build a prediction model with the terms from the best model in the previous search. In addition, we augment that model with adding the time ranges.

```
## Best model
mod_fit_disA_best <- train(A_dis_f~poly(mean.map,2)+AIS_ad+Injury_level+range_76_117, 
                 data = prediction.df.f_dis, method="glm", family="binomial",
                 trControl = ctrl,na.action = 'na.omit')

#Prediction probability
prob_predict_disA_best<-mod_fit_disA_best$pred[,3]

model_prediction_disA_best<-prediction_fun(prob_predict_disA_best,
                        prediction.df.f_dis$A_dis,ref = "1")

## noMap
mod_fit_disA_best_noMap <- train(A_dis_f~AIS_ad+Injury_level, 
                 data = prediction.df.f_dis, method="glm", family="binomial",
                 trControl = ctrl,na.action = 'na.omit')

#Prediction probability
prob_predict_disA_best_noMap<-mod_fit_disA_best_noMap$pred[,3]

model_prediction_disA_best_noMap<-prediction_fun(prob_predict_disA_best_noMap,
                        prediction.df.f_dis$A_dis,ref = "1")

model_prediction_disA_best$conf_mat
```

```
## Confusion Matrix and Statistics
## 
##           Reference
## Prediction  0  1
##          0 51  6
##          1 10 26
##                                           
##                Accuracy : 0.828           
##                  95% CI : (0.7357, 0.8983)
##     No Information Rate : 0.6559          
##     P-Value [Acc > NIR] : 0.0001856       
##                                           
##                   Kappa : 0.6299          
##                                           
##  Mcnemar's Test P-Value : 0.4532547       
##                                           
##             Sensitivity : 0.8125          
##             Specificity : 0.8361          
##          Pos Pred Value : 0.7222          
##          Neg Pred Value : 0.8947          
##              Prevalence : 0.3441          
##          Detection Rate : 0.2796          
##    Detection Prevalence : 0.3871          
##       Balanced Accuracy : 0.8243          
##                                           
##        'Positive' Class : 1               
##
```

```
model_prediction_disA_best_noMap$conf_mat
```

```
## Confusion Matrix and Statistics
## 
##           Reference
## Prediction  0  1
##          0 44 10
##          1 17 22
##                                           
##                Accuracy : 0.7097          
##                  95% CI : (0.6064, 0.7992)
##     No Information Rate : 0.6559          
##     P-Value [Acc > NIR] : 0.1631          
##                                           
##                   Kappa : 0.3886          
##                                           
##  Mcnemar's Test P-Value : 0.2482          
##                                           
##             Sensitivity : 0.6875          
##             Specificity : 0.7213          
##          Pos Pred Value : 0.5641          
##          Neg Pred Value : 0.8148          
##              Prevalence : 0.3441          
##          Detection Rate : 0.2366          
##    Detection Prevalence : 0.4194          
##       Balanced Accuracy : 0.7044          
##                                           
##        'Positive' Class : 1               
##
```

```
coef(mod_fit_disA_best$finalModel)
```

```
##          (Intercept) `poly(mean.map, 2)1` `poly(mean.map, 2)2` 
##          20.46637523          27.03166802         -17.13847834 
##              AIS_adA              AIS_adB              AIS_adC 
##         -22.81461535         -20.38324341         -19.00704633 
##              AIS_adD    Injury_levelOther         range_76_117 
##           0.21798245          -1.22893538           0.01777713
```

### 9.2.2 Predicting D discharge

#### 9.2.2.1 Search for best model

Search for the best model as for AIC. Single terms and pairwise interaction are allowed.

```
model_search_disD<-glmulti(D_dis~poly(mean.map,2)+mean.HR+length_surg+ days_post_surgery_dish+Age+AIS_ad+Injury_level+range_76_104+range_76_117, 
                 data = prediction.df.f_dis,family="binomial", level = 1,
                 crit = "aicc", plotty = F, report = F)

## Extract the model that minimizes AIC
best_model_disD<-model_search_disD@objects[[1]]
```

#### 9.2.2.2 Best model

```
best_model_disD
```

```
## 
## Call:  fitfunc(formula = as.formula(x), family = "binomial", data = data)
## 
## Coefficients:
## (Intercept)      AIS_adA      AIS_adB      AIS_adC      AIS_adD  length_surg  
##   -1.558670    -2.324926    -0.410017     2.591414     2.624289     0.004409  
##         Age  
##   -0.030004  
## 
## Degrees of Freedom: 92 Total (i.e. Null);  86 Residual
## Null Deviance:       115.4 
## Residual Deviance: 62.28     AIC: 76.28
```

#### 9.2.2.3 Prediction models

Next we build a prediction model with the terms from the best model in the previous search.

```
## Best model
mod_fit_disD_best <- train(D_dis_f~AIS_ad+length_surg+Age, 
                 data = prediction.df.f_dis, method="glm", family="binomial",
                 trControl = ctrl,na.action = 'na.omit')

#Prediction probability
prob_predict_disD_best<-mod_fit_disD_best$pred[,3]

model_prediction_disD_best<-prediction_fun(prob_predict_disD_best,
                        prediction.df.f_dis$D_dis,ref = "1")

## add MAP
mod_fit_disD_best_addMap <- train(D_dis_f~poly(mean.map, 2)+AIS_ad+length_surg+Age, 
                 data = prediction.df.f_dis, method="glm", family="binomial",
                 trControl = ctrl,na.action = 'na.omit')

#Prediction probability
prob_predict_disD_best_addMap<-mod_fit_disD_best_addMap$pred[,3]

model_prediction_disD_best_addMap<-prediction_fun(prob_predict_disD_best_addMap,
                        prediction.df.f_dis$D_dis,ref = "1")

model_prediction_disD_best$conf_mat
```

```
## Confusion Matrix and Statistics
## 
##           Reference
## Prediction  0  1
##          0 52  6
##          1 12 23
##                                           
##                Accuracy : 0.8065          
##                  95% CI : (0.7115, 0.8811)
##     No Information Rate : 0.6882          
##     P-Value [Acc > NIR] : 0.007487        
##                                           
##                   Kappa : 0.5732          
##                                           
##  Mcnemar's Test P-Value : 0.238593        
##                                           
##             Sensitivity : 0.7931          
##             Specificity : 0.8125          
##          Pos Pred Value : 0.6571          
##          Neg Pred Value : 0.8966          
##              Prevalence : 0.3118          
##          Detection Rate : 0.2473          
##    Detection Prevalence : 0.3763          
##       Balanced Accuracy : 0.8028          
##                                           
##        'Positive' Class : 1               
##
```

```
model_prediction_disD_best_addMap$conf_mat
```

```
## Confusion Matrix and Statistics
## 
##           Reference
## Prediction  0  1
##          0 50  7
##          1 14 22
##                                           
##                Accuracy : 0.7742          
##                  95% CI : (0.6758, 0.8545)
##     No Information Rate : 0.6882          
##     P-Value [Acc > NIR] : 0.04365         
##                                           
##                   Kappa : 0.5064          
##                                           
##  Mcnemar's Test P-Value : 0.19043         
##                                           
##             Sensitivity : 0.7586          
##             Specificity : 0.7812          
##          Pos Pred Value : 0.6111          
##          Neg Pred Value : 0.8772          
##              Prevalence : 0.3118          
##          Detection Rate : 0.2366          
##    Detection Prevalence : 0.3871          
##       Balanced Accuracy : 0.7699          
##                                           
##        'Positive' Class : 1               
##
```

```
coef(mod_fit_disD_best$finalModel)
```

```
##  (Intercept)      AIS_adA      AIS_adB      AIS_adC      AIS_adD  length_surg 
##  1.558669518  2.324926285  0.410017115 -2.591413887 -2.624289398 -0.004408631 
##          Age 
##  0.030003943
```

## 9.3 Figure 5

```
sense_spec_plot_improve<-model_prediction_best$sense_spec_plot+
  ggtitle("AIS impro.")

sense_spec_plot_disA<-model_prediction_disA_best$sense_spec_plot+
  ggtitle("A at disch.")

sense_spec_plot_disD<-model_prediction_disD_best$sense_spec_plot+
  ggtitle("D at disch.")

# ROC plot
auc_improve<-model_prediction_best$auc
auc_disA<-model_prediction_disA_best$auc
auc_disD<-model_prediction_disD_best$auc

roc_df_improve<-model_prediction_best$roc_df
roc_df_disA<-model_prediction_disA_best$roc_df
roc_df_disD<-model_prediction_disD_best$roc_df

roc_plot<-ggplot(roc_df_improve, aes(1-spec_y, sens_y))+
  geom_line(data=roc_df_disA, aes(1-spec_y, sens_y), color="green2")+
  geom_line(data=roc_df_disD, aes(1-spec_y, sens_y), color="steelblue1")+
  geom_line(color="firebrick")+
  theme_minimal()+
  scale_x_continuous(breaks=seq(0,1,0.25), 
                     labels = c("1", "0.75","0.5","0.25","0"))+
  xlab("Specificity")+
  ylab("Sensitivity")+
  geom_segment(aes(x=0, xend=1, y=0, yend=1), linetype="dashed")+
  
  annotate("text", label=paste("AIS impro. =",round(auc_improve, 3)),x=0.66, y=0.16)+
  geom_segment(aes(x=0.3, xend=0.36, y=0.15, yend=0.15), color="firebrick")+
  
  annotate("text", label=paste("A at disch. =",round(auc_disA, 3)),
           x=0.66, y=0.1)+
  geom_segment(aes(x=0.3, xend=0.36, y=0.1, yend=0.1), color="green2")+
  
  annotate("text", label=paste("D at disch. =",round(auc_disD, 3)),
           x=0.66, y=0.04)+
  geom_segment(aes(x=0.3, xend=0.36, y=0.03, yend=0.03), color="steelblue1")+
  #Annotate points of best model
  annotate("point", x=1-roc_df_improve$spec_y[which.min(roc_df_improve$diff_y)],
           y=roc_df_improve$sens_y[which.min(roc_df_improve$diff_y)], color="firebrick",
           size=4, alpha=0.3)+
  annotate("point", x=1-roc_df_disA$spec_y[which.min(roc_df_disA$diff_y)],
           y=roc_df_disA$sens_y[which.min(roc_df_disA$diff_y)],color="green2",
           size=4, alpha=0.3)+
  annotate("point", x=1-roc_df_disD$spec_y[which.min(roc_df_disD$diff_y)],
           y=roc_df_disD$sens_y[which.min(roc_df_disD$diff_y)],color="steelblue1",
           size=4, alpha=0.3)+
  annotate("point", x=0.05, y=0.94,color="grey", size=4, alpha=0.5)+
  annotate("text", label = "Threshold", x=0.22, y=0.95)

## Compose the model prediction figure
figure5<-(sense_spec_plot_improve+sense_spec_plot_disA+sense_spec_plot_disD+
            roc_plot+ggtitle("ROC"))+
  plot_annotation(tag_levels = 'a')+plot_layout(ncol=2)

ggsave(figure5, file="figure 5.png", width = 8, height = 8)

figure5
```

# 10 Session info

```
sessionInfo()
```

```
## R version 4.1.0 (2021-05-18)
## Platform: x86_64-w64-mingw32/x64 (64-bit)
## Running under: Windows 10 x64 (build 19042)
## 
## Matrix products: default
## 
## locale:
## [1] LC_COLLATE=English_United States.1252 
## [2] LC_CTYPE=English_United States.1252   
## [3] LC_MONETARY=English_United States.1252
## [4] LC_NUMERIC=C                          
## [5] LC_TIME=English_United States.1252    
## 
## attached base packages:
## [1] grid      splines   stats     graphics  grDevices utils     datasets 
## [8] methods   base     
## 
## other attached packages:
##  [1] glmulti_1.0.8      leaps_3.1          rJava_1.0-4        webshot_0.5.2     
##  [5] png_0.1-7          patchwork_1.1.1    ROCR_1.0-11        viridis_0.6.1     
##  [9] viridisLite_0.4.0  boot_1.3-28        caret_6.0-88       glmnetUtils_1.1.8 
## [13] glmnet_4.1-2       Matrix_1.3-3       table1_1.4.2       cccd_1.5          
## [17] TDAstats_0.4.1     RColorBrewer_1.1-2 vegan_2.5-7        lattice_0.20-44   
## [21] permute_0.9-5      visNetwork_2.0.9   reshape2_1.4.4     forcats_0.5.1     
## [25] stringr_1.4.0      dplyr_1.0.7        purrr_0.3.4        readr_1.4.0       
## [29] tidyr_1.1.3        tibble_3.1.2       ggplot2_3.3.5      tidyverse_1.3.1   
## [33] igraph_1.2.6      
## 
## loaded via a namespace (and not attached):
##  [1] colorspace_2.0-2     deldir_0.2-10        ellipsis_0.3.2      
##  [4] class_7.3-19         fs_1.5.0             rstudioapi_0.13     
##  [7] proxy_0.4-26         farver_2.1.0         prodlim_2019.11.13  
## [10] fansi_0.5.0          lubridate_1.7.10     xml2_1.3.2          
## [13] codetools_0.2-18     knitr_1.33           Formula_1.2-4       
## [16] jsonlite_1.7.2       pROC_1.17.0.1        broom_0.7.8         
## [19] cluster_2.1.2        dbplyr_2.1.1         compiler_4.1.0      
## [22] httr_1.4.2           backports_1.2.1      assertthat_0.2.1    
## [25] cli_3.0.0            htmltools_0.5.1.1    tools_4.1.0         
## [28] gtable_0.3.0         glue_1.4.2           Rcpp_1.0.7          
## [31] cellranger_1.1.0     jquerylib_0.1.4      vctrs_0.3.8         
## [34] ape_5.5              nlme_3.1-152         iterators_1.0.13    
## [37] timeDate_3043.102    xfun_0.24            gower_0.2.2         
## [40] ps_1.6.0             rvest_1.0.0          lifecycle_1.0.0     
## [43] MASS_7.3-54          scales_1.1.1         ipred_0.9-11        
## [46] hms_1.1.0            parallel_4.1.0       yaml_2.2.1          
## [49] gridExtra_2.3        sass_0.4.0           rpart_4.1-15        
## [52] stringi_1.6.2        highr_0.9            foreach_1.5.1       
## [55] e1071_1.7-7          lava_1.6.9           shape_1.4.6         
## [58] rlang_0.4.11         pkgconfig_2.0.3      evaluate_0.14       
## [61] labeling_0.4.2       recipes_0.1.16       htmlwidgets_1.5.3   
## [64] processx_3.5.2       tidyselect_1.1.1     plyr_1.8.6          
## [67] magrittr_2.0.1       R6_2.5.0             generics_0.1.0      
## [70] DBI_1.1.1            pillar_1.6.1         haven_2.4.1         
## [73] withr_2.4.2          mgcv_1.8-35          survival_3.2-11     
## [76] nnet_7.3-16          modelr_0.1.8         crayon_1.4.1        
## [79] utf8_1.2.1           rmarkdown_2.9        readxl_1.3.1        
## [82] data.table_1.14.0    callr_3.7.0          FNN_1.1.3           
## [85] ModelMetrics_1.2.2.2 reprex_2.0.0         digest_0.6.27       
## [88] stats4_4.1.0         munsell_0.5.0        bslib_0.2.5.1
```
